# How imputation can mitigate SNP ascertainment Bias

**DOI:** 10.1186/s12864-021-07663-6

**Published:** 2021-05-12

**Authors:** Johannes Geibel, Christian Reimer, Torsten Pook, Steffen Weigend, Annett Weigend, Henner Simianer

**Affiliations:** 1 Department of Animal Sciences, Animal Breeding and Genetics Group, University of Goettingen, Albrecht-Thaer-Weg 3, 37075 Göttingen, Germany; 2 Center for Integrated Breeding Research, University of Goettingen, Albrecht-Thaer-Weg 3, 37075, Göttingen, Germany; 3 Institute of Farm Animal Genetics, Friedrich-Loeffler-Institut, Höltystrasse 10, 31535 Neustadt-Mariensee, Germany

**Keywords:** SNP ascertainment bias, Imputation, Chickens, Population genetics

## Abstract

**Background:**

Population genetic studies based on genotyped single nucleotide polymorphisms (SNPs) are influenced by a non-random selection of the SNPs included in the used genotyping arrays. The resulting bias in the estimation of allele frequency spectra and population genetics parameters like heterozygosity and genetic distances relative to whole genome sequencing (WGS) data is known as SNP ascertainment bias. Full correction for this bias requires detailed knowledge of the array design process, which is often not available in practice. This study suggests an alternative approach to mitigate ascertainment bias of a large set of genotyped individuals by using information of a small set of sequenced individuals via imputation without the need for prior knowledge on the array design.

**Results:**

The strategy was first tested by simulating additional ascertainment bias with a set of 1566 chickens from 74 populations that were genotyped for the positions of the Affymetrix Axiom™ 580 k Genome-Wide Chicken Array. Imputation accuracy was shown to be consistently higher for populations used for SNP discovery during the simulated array design process. Reference sets of at least one individual per population in the study set led to a strong correction of ascertainment bias for estimates of expected and observed heterozygosity, Wright’s Fixation Index and Nei’s Standard Genetic Distance. In contrast, unbalanced reference sets (overrepresentation of populations compared to the study set) introduced a new bias towards the reference populations. Finally, the array genotypes were imputed to WGS by utilization of reference sets of 74 individuals (one per population) to 98 individuals (additional commercial chickens) and compared with a mixture of individually and pooled sequenced populations. The imputation reduced the slope between heterozygosity estimates of array data and WGS data from 1.94 to 1.26 when using the smaller balanced reference panel and to 1.44 when using the larger but unbalanced reference panel. This generally supported the results from simulation but was less favorable, advocating for a larger reference panel when imputing to WGS.

**Conclusions:**

The results highlight the potential of using imputation for mitigation of SNP ascertainment bias but also underline the need for unbiased reference sets.

**Supplementary Information:**

The online version contains supplementary material available at 10.1186/s12864-021-07663-6.

## Background

To perform cost- and computationally efficient, many of the population genetic studies of the last 10 years for humans [1, 2], as well as for model- [3, 4] and agricultural species [5–8] were based on single nucleotide polymorphisms (SNP), which were genotyped by commercially available SNP arrays. Those arrays are based on a non-random selection (ascertainment) of SNPs, and come with a bias relative to whole genome re-sequencing (WGS) data, widely known as SNP Ascertainment Bias [9–11].

To design an array, SNPs initially need to be discovered in a finite set of sequenced individuals, the discovery panel. The chance to discover globally common SNPs is higher in this finite set of individuals than the chance to discover globally rare SNPs. This results in allele frequency spectra of arrays showing a shift towards common SNPs as compared to allele frequency spectra of WGS, which typically contain a high share of rare SNPs [12]. Additionally, the discovery panel is typically not a random sample from the global population of a species, but over-represents individuals from more intensively researched populations, e.g. humans of Yoruban, Japanese, Chinese and European descent [13], commercially bred taurine cattle breeds [14] or commercial layer and broiler chicken lines [15]. SNPs that are common in those discovery populations are not necessarily globally common. As a consequence, allele frequency spectra of discovery populations are systematically skewed towards higher minor allele frequencies (MAF) than those of non-discovery populations [12, 16]. In extreme cases, e.g. when used for samples of other species, this can result in a lack of variable and thus informative SNPs on the array and therefore a shift of the frequency spectrum towards rare variants [16].

The shift in the allele frequency spectra has an effect on population genetic estimators that depend on the allele frequency estimates. Exemplarily, the shift in allele frequencies towards common variants leads to an systematic overestimation of the heterozygosity of populations [16, 17]. The relative effect is stronger for populations that were part of the discovery set compared to populations that were not part of the discovery set [16]. Since commercially used breeds tend to be overrepresented in discovery sets [14, 15], their diversity thus tends to be overestimated compared to non-commercial breeds not included in the discovery set [16]. Systematic differences in allele frequency spectra further increase estimates of genetic distances between populations which were part of the discovery set and those which were not [10].

The complex interaction between the size of the discovery panel and its restriction to a subset of populations makes it difficult to predict or outright correct for the effect of SNP ascertainment bias. Further, attempts to implement bias-reduced estimators require strong assumptions on the design process of the used SNP array [12], which is often not public knowledge or too complicated to be remodeled [18, 19]. Malomane et al. [17] therefore screened different raw data filtering strategies on mitigation of ascertainment bias in SNP data and identified linkage pruning to result in slightly decreasing ascertainment bias. Due to strongly decreasing sequencing costs and the complexity of the ascertainment bias correction strategies, more and more studies started using WGS data for population genetic analysis during the last years [20–24]. However, costs for broad WGS based studies are still rather high, resulting in large-scale collaborations such as the 1000 Genomes Project [25], the 1000 Bull Genomes Project [26], or the 1001 Arabidopsis Genomes Project [27].

A commonly used method to in silico increase the resolution of SNP data sets is imputation [28]. Over the years a variety of imputation approaches [29–35] have been proposed that utilize linkage, pedigree, and haplotype information. To increase the marker density, an additional reference panel of individuals that were genotyped/sequenced by the intended resolution is required to additionally infer information from SNPs missing on the respective lower density study set.

Imputation-based studies mostly either used a reference panel of the same population as the study set itself [36–38] or utilized large global reference panels such as the 1000 Genomes [25, 39, 40] or 1000 Bull genomes [26, 41] projects. Especially for admixed or small endangered populations, the use of additional distantly related populations in the reference panel was investigated. On one hand, Brøndum et al. [42], Ye et al. [43] and Rowan et al. [44] identified multi-breed reference panels to increase imputation accuracy especially in admixed breeds and for low frequent alleles when imputing from high-density genotypes to sequence data. On the other hand, Berry et al. [45] observed that smaller within breed reference panels (140–688 reference cattle individuals per breed) performed always superior compared to the combined across breed reference panel when imputing from low density to high-density array genotypes. Korkuć et al. [46] showed that adding 100 to 500 Holstein cattle sequences to a reference panel of 30 German Black Pied cattle significantly decreased the imputation accuracy in comparison to the pure panel when imputing from array to sequence data. Adding the same numbers of a multi-breed reference panel only outperformed the pure panel when at least 300 reference animals were added. Pook et al. [47] investigated the inclusion of chicken populations to the reference set which were differently distantly related to the study set. While error rates generally decreased for rare alleles, the inclusion of distantly related populations slightly increased error rates for previously good imputed SNPs. Overall, the ideal setup of a reference panel seems to be highly dependent on the application with positive effects for some, but also potential harm in other cases.

In this context, the current study aims at assessing the influence of a study design on SNP ascertainment bias, which uses a small number of sequenced chickens (the reference set) to in silico correct SNP ascertainment bias in a broad multi-population set of genotyped chickens (the study set) by imputation to sequence level. The general idea behind this design is to allow for a large sample size, which reduces sampling bias while keeping sequencing costs affordable as most individuals will only be genotyped. We, therefore, assessed the potential effects of this design by imputing in silico created low-density array data to high-density array data, and by imputing real high-density data to WGS data.

## Material and methods

### Data

Three different sets of genomic data were used for this study:

Set 1: Individual sequence data of 68 chickens from 68 different populations, sequenced within the scope of the EU project Innovative Management of Animal Genetic Resources (IMAGE; www.imageh2020.eu) [48]. They were complemented by 25 sequences (17 + 8) from two commercial white layer lines, 25 sequences (19 + 6) from two commercial brown layer lines, and 40 sequences (20 each) from two commercial broiler lines [23]. In total 158 sequences from 74 populations.

Set 2: Pooled sequence data from 37 populations (9–11 chickens per population) [17]. All except 4 chickens from two populations were part of set 3.

Set 3: Genotypes of 1566 chickens from 74 populations, either genotyped (sub-set of the Synbreed Chicken Diversity Panel; SCDP) [49] with the Affymetrix Axiom™ 580 k Genome-Wide Chicken Array [15], or complemented from set 1.

**Fig. 1:**
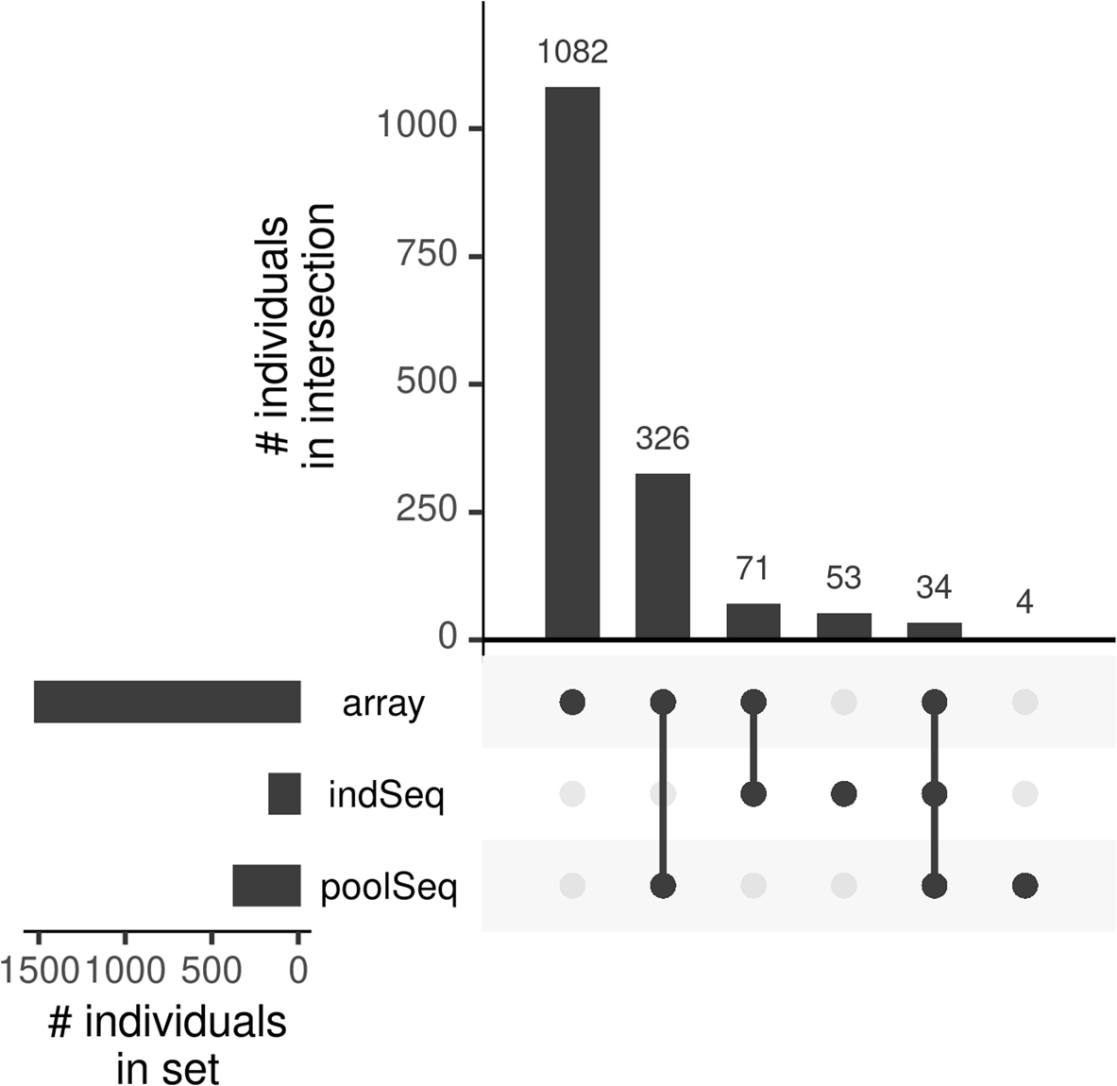
UpSet plot showing the distinct intersections of chickens between the used sequencing/ genotyping technologies. The left bar plot contains the total number of individuals that were genotyped (array), individually sequenced (indSeq), or pooled sequenced (poolSeq). The upper bar plot contains the number of individuals within each distinct intersection, indicated by the connected points below

The intersection of the used data sets is shown in Fig. 1 and accession information of the raw data per sample can be found in Supplementary File 1. All three data sets came with their own characteristics. While individual sequences are considered to be the gold standard throughout this study, genotypes of the Affymetrix Axiom™ 580 k Genome-Wide Chicken Array [15] are biased towards variation which is common in the commercial chicken lines [16] and pooled sequences only allow for an estimate of population allele frequencies and show a slight bias due to sample size and coverage (Supplementary File 2) [50, 51].

### Calling of WGS SNPs and generation of genotype set

Alignment of the raw sequencing reads against the latest chicken reference genome GRCg6a [52] and SNP calling was conducted for individual and pooled sequenced data following GATK best practices [53, 54]. As the Affymetrix Axiom™ 580 k Genome-Wide Chicken Array [15] does not contain enough SNPs on chromosomes 30–33 for imputation (and chromosome 29 is not annotated in the reference genome), only up to chromosome 28 was used. This resulted in 20,829,081 biallelic SNPs on chromosomes 1–28 which were used in further analyses. Additionally, all individual sequences were genotyped for the positions of the Affymetrix Axiom™ 580 k Genome-Wide Chicken Array [15].

To ensure compatibility between Array- and WGS data, the genotypes of the Synbreed Chicken Diversity panel were lifted over from galGal5 to galGal6 and corrected for switches of reference and alternate alleles. Only SNPs with known autosomal position, call rates > 0.95 and genotype recall rates > 0.95 were further considered. MAF filters were later used when subsampling the different sets and thus not considered in this step. Further, missing genotypes were imputed using Beagle 5.0 [35] with ne = 1000 [47] and the genetic map taken from Groenen et al. [55]. This resulted in a final set of 1566 animals from 74 populations (18–37 animals per population) and 462,549 autosomal SNPs, further referred to as the genotype set.

As Malomane et al. [17] described LD-based pruning as an effective filtering strategy to minimize the impact of ascertainment bias in SNP array data, the genotype set was additionally LD pruned using plink 1.9 [56] with --indep 50 5 2 flag. This reduced the genotype set to 136,755 SNPs (30%) and will be referred to as pruned genotype set.

The description of the detailed pipeline can be found in Supplementary File 2.

### Analyses based on simulation of ascertainment bias within the genotype set

A first comparison was based solely on the 15,868 SNPs of chromosome 10 of the genotype set which allowed for a high number of repetitions while still being based on a sufficiently sized chromosome. To simulate an ascertainment bias of known strength, an even more strongly biased array was designed in silico from the genotype set for each of the 74 populations (further called discovery populations) by using only SNPs with MAF > 0.05 within the according discovery population. This simulates the limitation to common variants in the discovery samples, which is the main reason for the ascertainment bias. Then, reference samples for imputation were chosen in five different ways with 10 different numbers of reference samples and three repetitions per sampling:

1. allPop_74_740: Equally distributed across all populations by sampling one to 10 chickens per population (74–740 reference samples).
2. randSamp_5_50: 5, 10, …, 50 randomly sampled chickens (5–50 reference samples).
3. randPop_5_50: Five chickens from each of one to 10 randomly sampled populations (5–50 reference samples).
4. minPop_5_50: Five chickens from each of one to 10 populations which were closest related to the discovery population, based on Nei’s Distance ([57]; 5–50 reference samples).
5. maxPop_5_50: Five chickens from each of one to 10 populations which were most distantly related to the discovery population, based on Nei’s Distance ([57]; 5–50 reference samples).

**Fig. 2:**
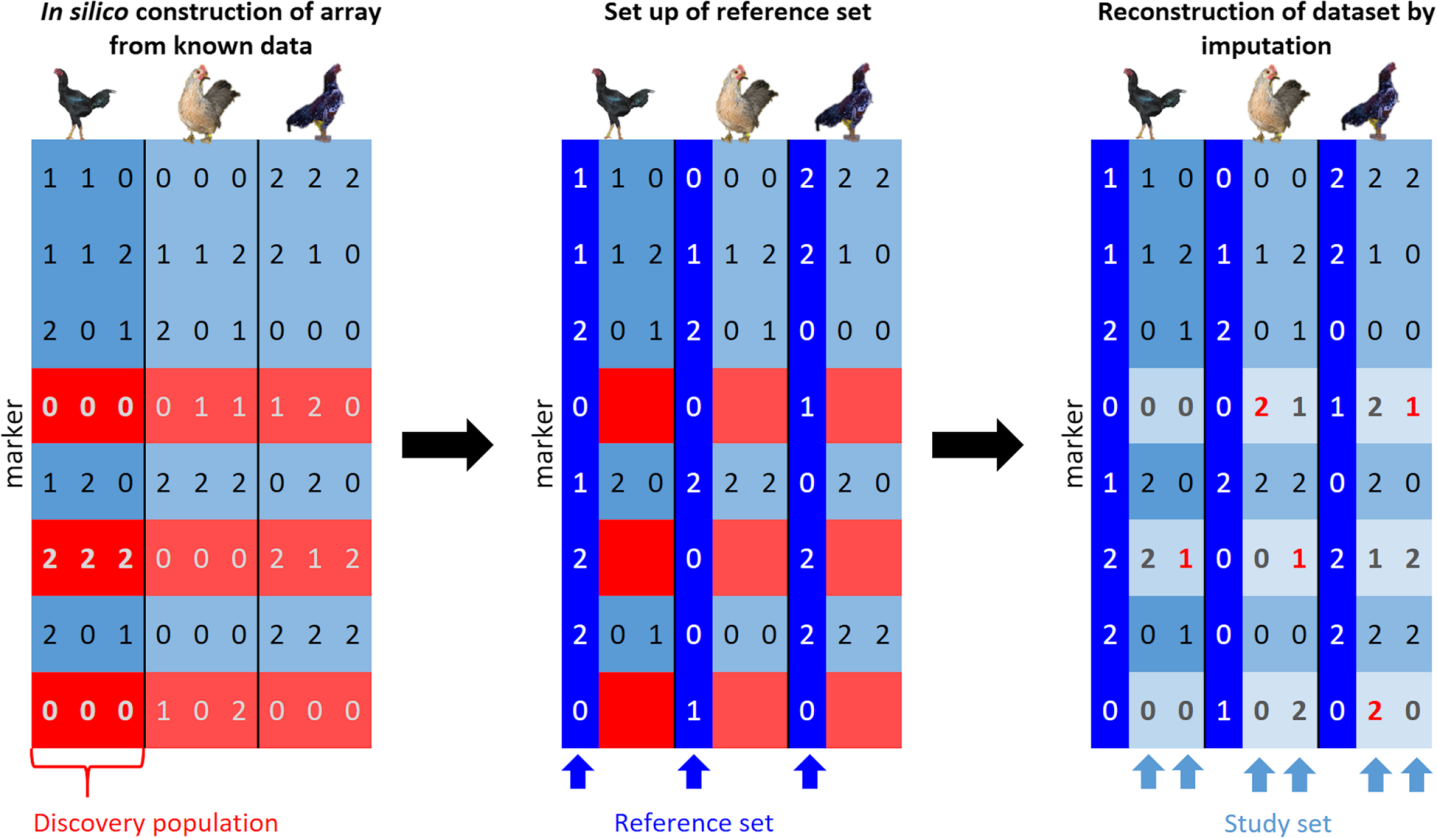
Schematic representation of the workflow of creating and re-imputing the in silico arrays. The starting point was a 0/1/2 coded marker matrix with SNPs in rows and individuals in columns (different populations separated by vertical lines). In a first step, an array (light blue rows) was constructed in silico from known data by setting all SNPs to missing which were invariable (MAF < 0.05, red rows) in the discovery population (first three columns). In a second step, a reference set (dark blue columns) was set up from animals for which complete knowledge of all SNPs was assumed. This Reference set was then used in a third step to impute the missing SNPs in the study set using Beagle 5.0 and resulting in a certain amount of imputation errors (red numbers)

This resulted in 2200 repetitions of in silico array development and re-imputation per sampling strategy. The reference set was formed by sub-setting the total genotype matrix to SNPs with MAF > 0.01 within the reference samples and the reference samples chosen via the above-mentioned strategies. Imputation of the in silico arrays to the reference set was performed by running Beagle 5.0 [35] with ne = 1000 [47], the genetic distances taken from Groenen et al. [55] and the according reference set. The schematic workflow can be found in Fig. 2.

Analyses were then based on comparisons between the in silico ascertained and later imputed sets and the genotype set, which was considered as the ‘true’ set for those comparisons.

### Imputation of genotype set to sequence level

After the initial tests of the imputation strategies by the in silico designed arrays, we imputed the complete genotype set to sequence level, using the available individual sequences as the reference panel. In the first run, one reference sample per sequenced population was chosen (74 reference samples; 74_1perLine) which is equivalent to the first scenario allPop_74 of the in silico array imputation. As we had more than one sequenced individual for the commercial lines, the number of reference samples for the commercial lines was subsequently increased to five reference samples per line (up to 98 reference samples; 98_5perLine). Finally, we used all available individually sequenced animals as reference samples (158 reference samples; 158_all), which resulted in a strong imbalance towards the two broiler lines (20 reference samples per broiler line).

Parameter settings in Beagle were further tweaked by increasing the window parameter to 200 cM to ensure enough overlap between reference and study SNPs. This was needed as we observed low assembly quality and insufficient coverage of the array on the small chromosomes. Analyses were then based on comparisons between the genotype set, the pruned set or the imputed sets and the gold standard, the WGS data.

### Comparison of population genetic estimators

Ascertainment bias shows its primary effect on the allele frequency spectrum. As populations are affected differently, we first concentrated on two heterozygosity estimates: expected (H_E_) and observed (H_O_) heterozygosity, which summarize per-population allele frequency spectra. We additionally included two allele frequency dependent distance measurements: Wright’s fixation index (F_ST_) [58] and Nei’s distance (D) [57].

H_O_, as the proportion of heterozygous genotypes in a population, could only be calculated when the genotypic status of a population was known (individual sequences or genotypes). In contrast, H_E_ could also be calculated from pooled sequences which allow the estimation of allele frequencies (p). Thereby, H_O_ and H_E_ (Eq. (1)) are calculated as average over all loci (l = 1, …, L).

1 $$ {H}_E=\frac{\sum \limits_l2{p}_l\left(1-{p}_l\right)}{L} $$

As pooled sequence data comes with a slight but systematic underestimation of H_E_ ([50]; Supplementary File 2), H_E_ for pooled sequences was multiplied with the correction factor $\frac{n}{n-1}$, introduced by Futschik and Schlötterer [50], where *n* is the number of haplotypes in the pool. This partially corrected the H_E_ estimates for the bias introduced by pooled sequencing (Supplementary File 2).

D was calculated as given by Eq. (2), where D_xy_ accounts for the genetic distance between populations X and Y, while x_il_ and y_il_ represent the frequency of the i^th^ allele at the l^th^ locus in population X and Y, respectively.

2 $$ {D}_{xy}=-\ln \left(\frac{\sum \limits_l\sum \limits_i{x}_{il}{y}_{il}}{\sqrt{\sum \limits_l\sum \limits_i{x}_{il}^2\sum \limits_l\sum \limits_i{y}_{il}^2}}\right) $$

Pairwise F_ST_ values between populations X and Y were estimated using Eq. (3), where *HT*_*l*_ accounts for the H_E_ within the total population at locus *l* and ${\overline{HS}}_l$ for the mean H_E_ within the two subpopulations at locus *l* [58].

3 $$ {F}_{ST}=\frac{\sum \limits_l\left({HT}_l-{\overline{HS}}_l\right)}{\sum \limits_l{HT}_l} $$

D and F_ST_ both show a downward bias that is comparable to HE when estimated from pooled data (Supplementary File 2). The effect of ascertainment bias is much larger than the effect of pooling for D. In contrast, F_ST_ is generally robust against the effects of ascertainment bias when a sufficiently large discovery panel was used for array development [10]. Therefore, it shows underestimation when calculated from pooled sequence data which is larger than the effect of ascertainment bias (Supplementary File 2). We therefore could not dissect the effects of the two biases in the comparisons on sequence level and did not include F_ST_ there.

Having no ascertainment bias would mean that estimates of a respective set would lie on the line of identity (diagonal) when regressing the set against the true values. The magnitude of the bias can therefore be defined as the distance of the estimates to that line. We therefore regressed the estimates from biased data (*y*_*ij*_) on the unbiased ones (*x*_*ij*_) while fitting group specific intercepts (*group*_*i*_) as well as group-specific slopes (*group*_*i*_ × *β*_*i*_) and a random error (ϵ_*ij*_, $\upepsilon \sim N\left(0,\mathbf{I}{\sigma}_e^2\right)$) as in Eq. (4).

4 $$ {y}_{ij}={group}_i+{group}_i\times {\beta}_i{x}_{ij}+{\upepsilon}_{ij} $$

The definition of a group describes for within-population estimators (e.g. H_E_) whether a population was used for SNP discovery (discovery population), samples from that population were used as reference set (reference population) or none of both (application population). Note that in scenarios where reference individuals were present for every population, we only divided them into discovery and application populations. For between population estimators (F_ST_, D), a group describes the according combination of the two involved population groups. Differences of the estimated slopes from one and the correlation between heterozygosity and distance estimates from biased and true set within groups were used as indicators for the magnitude of bias and random estimation error.

To get a measure for a fixed estimation error, we also calculated the mean overestimation across populations (j = 1 ... J) as in Eq. (5).

5 $$ mean\ \mathrm{overestimation}=\frac{\sum \limits_j\frac{biased\kern0.5em {estimate}_j- true\kern0.5em {estimate}_j}{true\kern0.5em {estimate}_j}}{J} $$

Note, that we had more than one (pooled) sequenced chicken for only 45 populations. Comparisons of population estimates on sequence level are therefore limited to 45 populations out of the 74 populations which were used as study and reference set for the imputation process.

### Assessment of imputation accuracy

Assessment of imputation accuracy was done by using Pearson correlation (r) between true and imputed genotypes [45, 59] for the in silico designed arrays. Pearson correlation puts a higher relative weight on imputation errors in rare alleles than plain comparison of allele- or genotype concordance rates [59]. In case of the imputation to sequence level, we used leave-one-out validation to assess per-animal imputation accuracy. However, the leave-one-out validation in our case shows a slightly downward biased accuracy estimate for the non-commercial samples (Figure S11, Supplementary File 2). For validation, the only sequenced sample of those populations was the test sample, which had to be removed from the reference set. Therefore, no closely related sample to the test sample remained in the reference set and the accuracy was subsequently underestimated. We additionally used the internal Beagle quality measure, the dosage r-squared (DR2) [60] to evaluate per-SNP imputation accuracy. This, however, only shows the theoretical imputation accuracy and cannot capture biases due to biased reference sets.

## Results

### In silico array to genotype

**Fig. 3:**
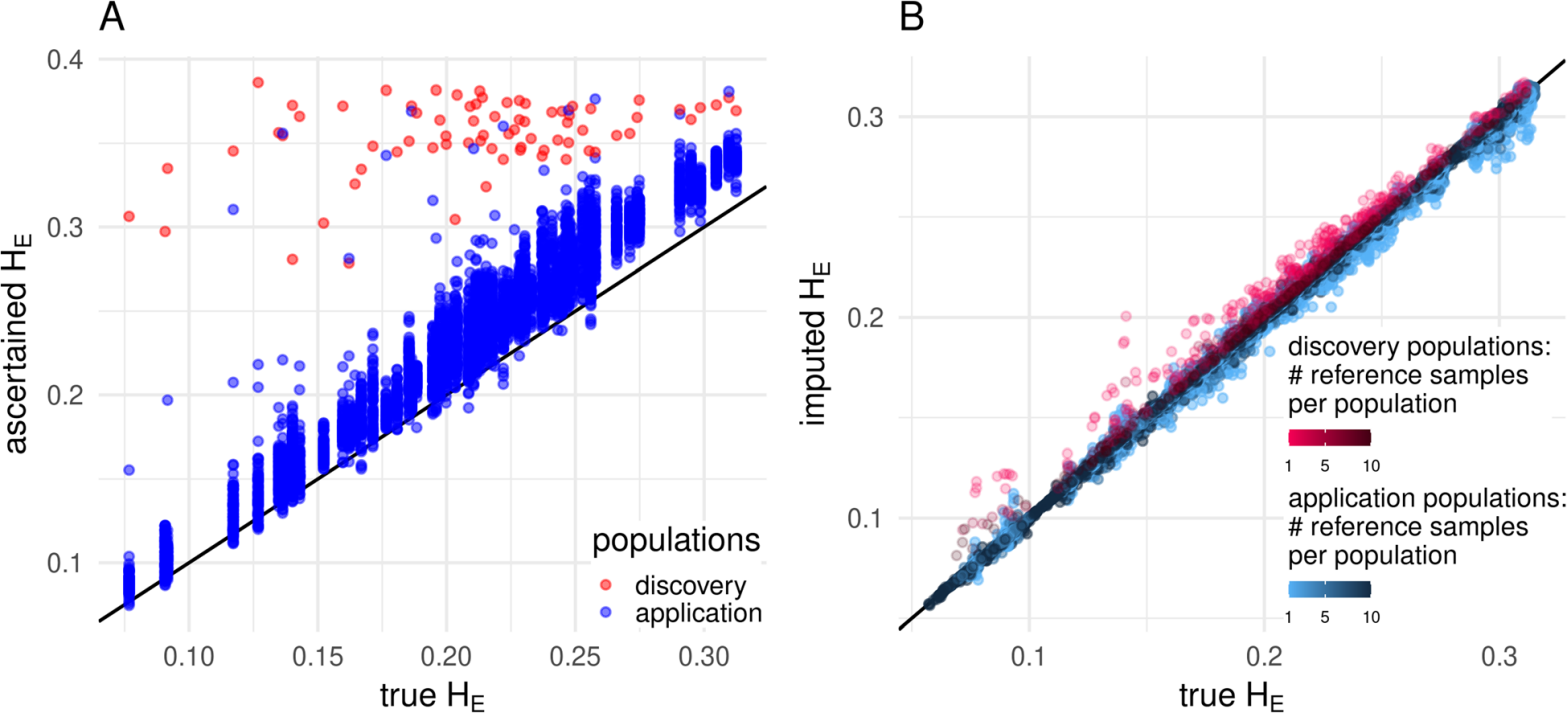
True H_E_ vs. ascertained H_E_ (**a**) and imputed H_E_ (**b**) by population group. For the imputed case, the strategy of using the same number of reference samples per population (allPop_74_740) is shown, an increase in the number of reference samples per population (1–10) is marked by an increasing color gradient and the line of identity is marked by a solid black line

As expected, the in silico ascertained sets showed a strong overestimation of the H_E_ for nearly all populations in all cases. The overestimation was much stronger for populations used for SNP discovery (Fig. 3a). Imputation using an equal number of reference samples per population (scenario allPop_74_740) massively decreased this bias (Fig. 3b). The correction became stronger with an increasing number of reference populations.

**Fig. 4:**
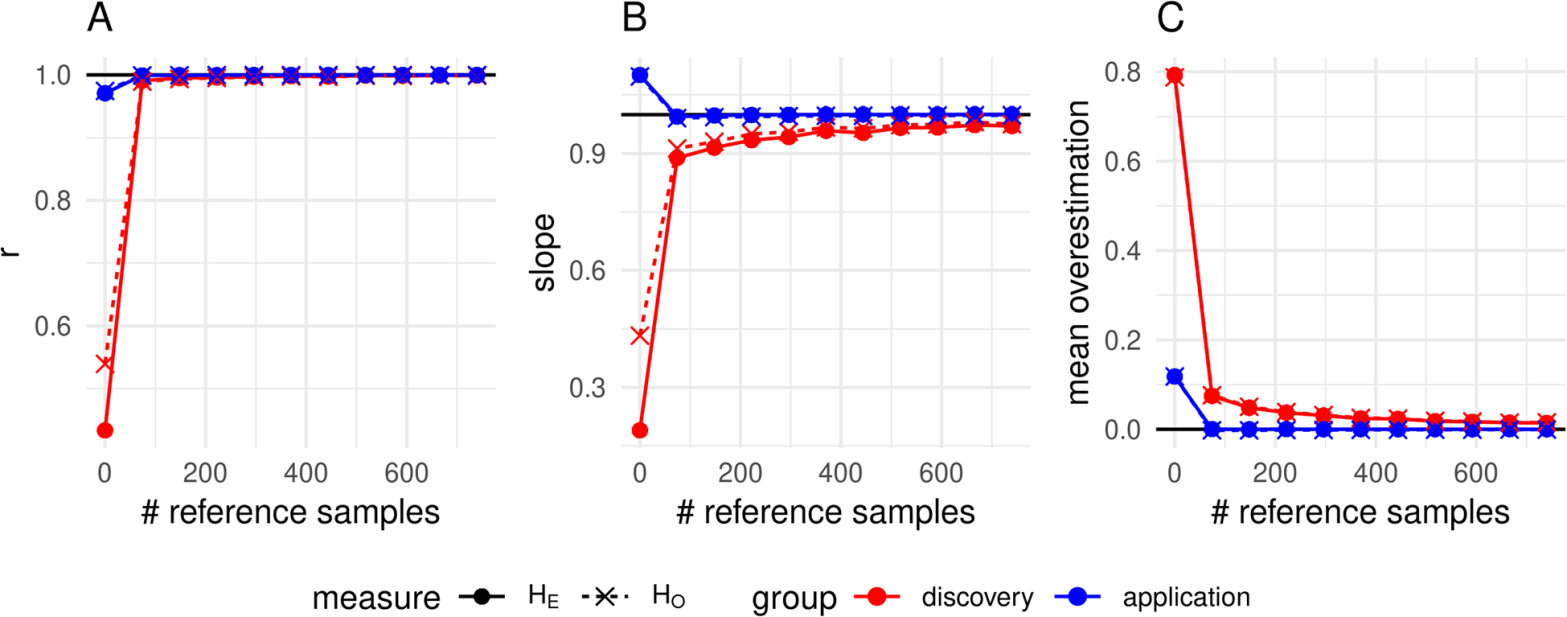
Development of correlation within population group (**a**), slope (**b**) and mean overestimation (**c**) of the regression lines for the two heterozygosity estimates when distributing the reference samples equally across all populations (allPop_74_740). The intended value for unbiasedness and minimum variance is marked as dense black horizontal line. Note that the case without imputation is consistent with zero reference samples

To get an impression on the strength of the correction and the needed size of the reference panel, Fig. 4 compares the correlation by population group, the slope for the within-group regression of the true H_E_ and H_O_ vs. the ascertained/ imputed cases and mean overestimation for strategy allPop_74_740. It shows that the effects of ascertainment bias were stronger for H_E_ than for H_O_. Imputation when using the reference set with just one individual per population corrects the initially much lower correlation within population group to > 0.99. While slope and mean overestimation are also pushed promptly towards the intended values of one and zero respectively for the non-discovery populations, there remains a small bias for the discovery populations, which decreases with an increasing number of reference samples.

The effects were observed in a comparable manner for the other imputation strategies (Figure S3). Due to smaller reference panels, the correction effect of the imputation was generally worse than for strategy allPop_74_740. Interestingly, when limiting the reference samples to a small number of populations (strategies randPop_5_50, minPop_5_50, maxPop_5_50), we observed a newly introduced bias towards the reference populations (Figure S3). This effect was strongest for strategy maxPop_5_50, where we chose the reference populations with a maximum distance from the discovery population. However, increasing the number of reference samples minimized the bias of reference and discovery populations with all strategies.

The effects of ascertainment bias were less pronounced in the distance measurements (D and F_ST_;Figure S4) than in the heterozygosity estimates. The bias was thereby only of numerical relevance, when estimating the distances between populations which belong to differently strongly biased population groups and was partly increased for some population groups by imputation with unbalanced reference samples (Figure S5). Note that F_ST_ was, all in all, less affected than D.

**Fig. 5:**
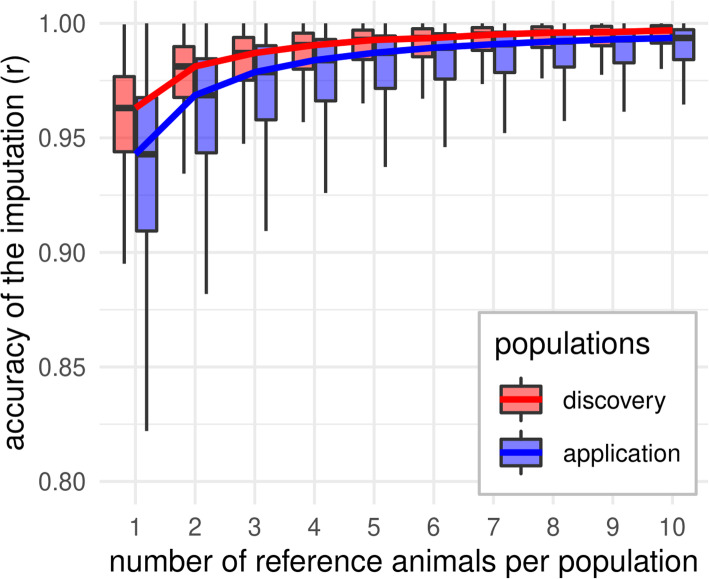
Development of the per-animal imputation accuracy for the in silico array to genotype set imputation with an increasing number of reference animals per population. Individuals are grouped on whether they belong to the population used for SNP discovery or not and reference individuals were chosen as in scenario allPop_74_740. The lines show the trend of the median and outliers are not shown in the plot as they do not add valuable information due to the high number of repetitions

The reduction of ascertainment bias was accompanied by high per-animal imputation accuracies (r). Strategy allPop_74 (one reference individual per population) resulted in a median imputation accuracy of 0.94. Increasing the number of reference individuals subsequently increased the accuracy up to 0.99 for 10 reference individuals per population (allPop_740). The accuracy was consistently higher for individuals which were part of the discovery population (Fig. 5). Accuracies were lower for the other strategies, mainly due to a maximum number of 50 reference individuals, which are fewer than the 74 of allPop_74. Detailed results for imputation accuracy can be found in Figure S2 and Supplementary File 2.

### Genotype to sequence

**Fig. 6:**
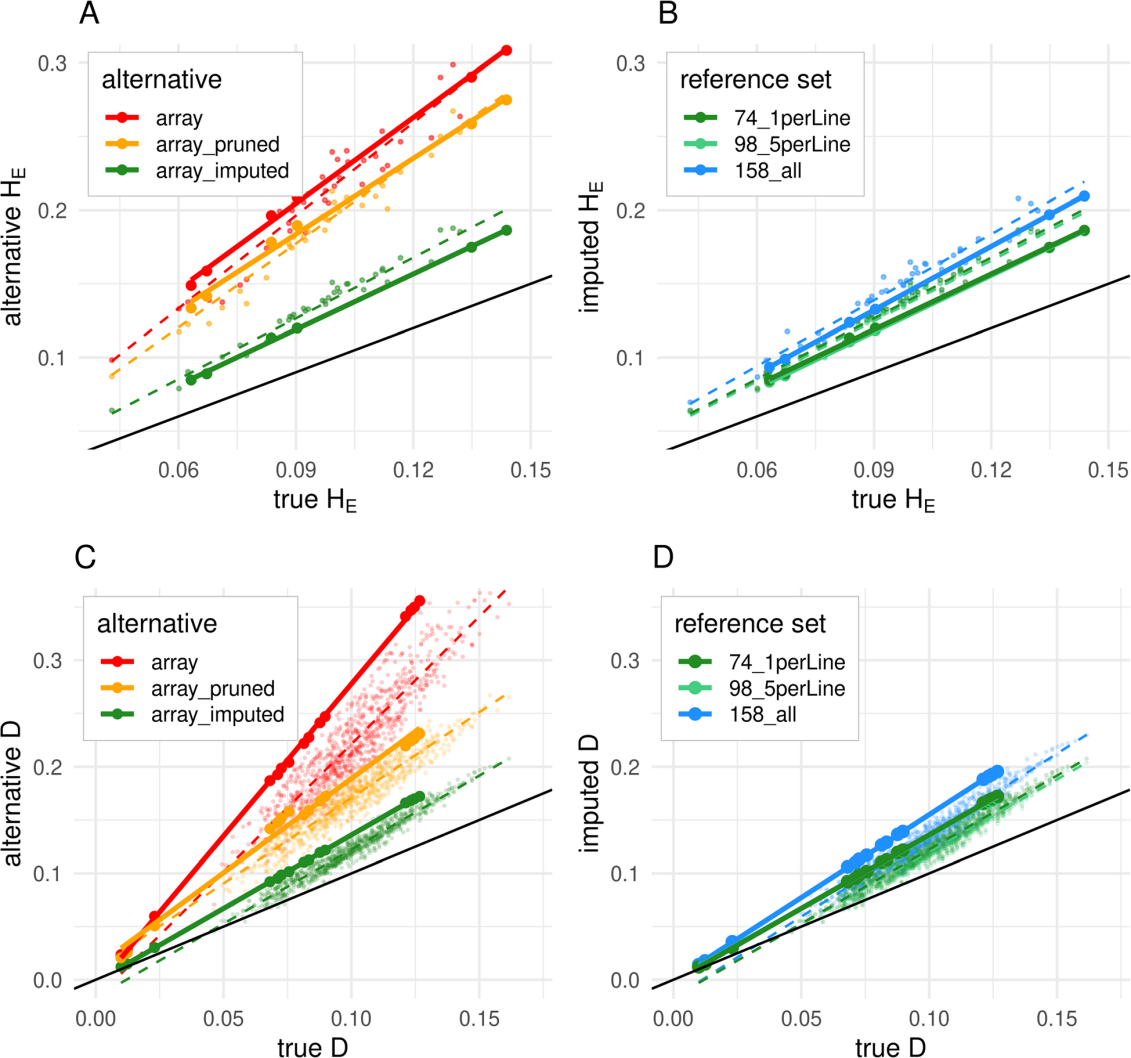
Effect of different correction strategies on ascertainment bias for expected heterozygosity (H_E_; A + B) and for Nei’s standard genetic distance (D; C + D). A + C – uncorrected array, linkage pruned array and imputed array (reference set 74_1perLine) based vs. sequence-based H_E_/ D. B + D – array imputed with different reference sets vs. sequence-based H_E_/ D. The solid black line represents the line of identity, the solid colored lines are regression lines within the individually sequenced populations (larger points) and the dashed lines regression lines within all populations which include individually and pooled (small points) sequenced populations. Note that there is also an effect of pooled sequencing which affects the ‘true’ values of the pooled sequenced populations

The effect of imputation to WGS on ascertainment bias of H_E_ is shown in Fig. 6. Given the situation that we cannot completely exclude pooling bias for the pooled sequenced samples (Supplementary File 2), only the effect on the individually sequenced samples can be discussed with adequate reliability. While the regression of array-based H_E_ estimates on sequence-based H_E_ estimates showed a slope of 1.94 for the individually sequenced populations, the linkage pruning slightly reduced this slope to 1.71. The clearly best result was achieved with imputation to WGS (slope = 1.26; 74_1perLine; Fig. 6a). This effect was also observed when considering all samples. However, note that there is also a slight effect of the remaining pooling bias, which cannot be separated from ascertainment bias for the pooled sequenced populations. Slightly increasing the reference panel (Fig. 6b) up to five samples per commercial line (98_5perLine) does not show any effect, while using all commercial samples in the reference panel (158_all) and thereby clearly biasing the reference panel towards the broiler samples increases HE again for all samples (slope = 1.44).

The results for Nei’s standard genetic distance (D;Fig. 6) showed the same pattern as the results for HE. The slope for distances between individually sequenced populations decreased from 2.86 (array) and 1.77 (array_pruned) to 1.38 (imputed, 74_1perLine). The unbalanced reference panel 158_all then again increased the slope to 1.56. The correlation for all distances, besides being also influenced by pooling bias and therefore being a rough estimate, was increased from 0.93 (array) respectively 0.95 (array_pruned) to 0.98 (all reference sets).

The overall imputation accuracy was lower than the one obtained for in silico array to array imputation. Increasing the number of commercial reference samples only resulted in increased imputation accuracies for the commercial samples. See Supplementary File 2, Table S1, Figure S6, Figure S7 and Figure S11 for details.

## Discussion

### Overall performance of the correction method

Imputation of SNP data sets from lower to higher density is a commonly used technique to either increase the resolution of data sets [36, 37, 41] or make them comparable across different platforms [61, 62]. The according studies mostly use a relatively homogeneous study set and a closely related and large reference set [36, 37]. However, studies exist which investigate the effect of increasing the reference set to a multi-population reference set to use an increased number of reference haplotypes [42–46]. To our knowledge, we here present the first study that investigates the use of a relatively small and diverse reference set on a large and diverse study set to correct for a genotyping platform-specific bias, the SNP ascertainment bias.

This approach intends that single imputation errors do not harm, if the mean across the genome, presented by different population genetic estimators, shows unbiased results with minimum variance. Therefore, imputation to WGS level using a comparably small reference panel can be used to correct for the ascertainment bias of commercial arrays.

Especially the in silico ascertained SNP arrays showed that even a very small reference panel consisting of one individual of each population showed very good results for all investigated estimators (e.g. correlation between biased H_E_ and true H_E_ of initially < 0.5 for the discovery populations increased to > 0.99; Fig. 4; Figure S4) and became better with an increasing number of reference populations. The results were less beneficial for the real WGS data, but also showed a strong decrease of the slope towards one. From the imputed in silico arrays, we could additionally realize a fast closing of the gap of the stronger overestimation of heterozygosity within discovery populations and the less severe overestimation in non-discovery populations. This also seemed to be the case when imputing to WGS level where we observed that the slope within the commercial populations (closely related to discovery populations of the real array) decreased more than the slope within all populations due to imputation. However, this observation in the WGS data has to be regarded with caution, as we additionally identified a non-negligible bias due to pooled sequencing which interfered with the assessment of ascertainment bias and which was, in our study, confounded with the difference between commercial populations (sequenced individually) and non-commercial populations (sequenced as pools).

The use of WGS information via imputation also consistently showed better results in regard of reduction of ascertainment bias than using linkage pruned array SNPs which was reported to be an effective filtering strategy for ascertainment bias mitigation by Malomane et al. [17].

Generally, the effect of imputation on the investigated estimators was shown to be comparable across estimators, regardless of their initial reaction to ascertainment bias. An interesting side observation was that F_ST_ did not show any ascertainment bias on the real array data (Figure S10) when calculated in the form of summing the numerator across SNPs and dividing by the sum of the denominator as calculated in this study. F_ST_ was only affected when used to estimate differentiation between the discovery- and non-discovery populations in the simulated array data, whose heterozygosity estimates were affected by ascertainment bias to a different degree. This strongly supports the findings of Albrechtsen et al. [10], who showed F_ST_ to be relatively robust against the effects of ascertainment bias.

We also investigated the effect of differently sized and constructed reference sets for imputation. Generally, larger reference sets increased the accuracy of imputation and thus decreased the ascertainment bias more than smaller reference sets. The best results were achieved when the reference set was as evenly distributed across the study set as possible. When reference populations were closely related to the discovery population, reduction in imputation quality and increase in ascertainment bias were less severe in case of unbalanced reference sets than if distantly related reference populations were used. This suggests that variation within study- and reference set needs to show enough overlap to achieve sufficient imputation accuracy and therefore reduction of ascertainment bias.

Results from literature suggest that multi-breed reference panels generally increase imputation accuracy especially for rare variants and within admixed populations [42–44]. Additionally, Rowan et al. [44] argue that they do not seem to introduce variation at a relevant scale for markers for which the breeds are actually fixed. However, some studies also showed that strongly unbalanced reference sets can reduce imputation accuracy [45, 46]. In this study, including additional reference samples in a biased way when going from reference set 74_1perLine to 158_all increased the effects of ascertainment bias on H_E_ and D. Additionally, only the commercial populations, for which we increased the number of reference samples, showed a gain in per-animal imputation accuracy (Figure S11). However, theoretical imputation accuracies rather increased than decreased (Figure S6; Table S1) for previously poorly imputed SNPs. The increase in accuracies for poorly imputed SNPs supports the findings of Brøndum [42], Rowan et al. [44] and Ye et al. [43] that multi-breed reference panels rather help in getting better imputation results. However, the missing gain in per-animal accuracy for non-commercial populations together with the observed bias in the leave-one-out validation for our sparse reference set highlights the still existing need for closely related individuals as shown by Berry et al. [45], Korkuć et al. [46] and Pook et al. [47]. The worsening effect on bias correction, however, highlights the main reason for ascertainment bias. One can only identify variation which is present in the investigated samples. When developing an array, one observes the variation in the discovery set, while in our case we observed variation in the reference set used for imputation. An overrepresentation of certain populations in the reference set biases estimators towards variation present in those populations. Besides the aforementioned effects in the imputations to WGS, we also observed this by an increasing bias for the unbalanced reference sets in the in silico array imputations (Figure S3, Figure S5). Therefore, it is crucial to use a reference set for imputation which covers the intended range of variation.

Besides the previously described effects of imputation on ascertainment bias, we also identified an effect of array design on imputation accuracy. Discovery populations show higher imputation accuracies than non-discovery populations (Figure 5). As markers on arrays are more representative for discovery populations than non-discovery populations, relatively more of the genetic variability in discovery populations is explained by the array and imputation is more accurate on average.

## Conclusion

The problem to which we provide at least a partial solution is that relevant population genetic parameters are systematically biased through the design process of SNP arrays. Imputation was able to mitigate this SNP ascertainment bias in our samples for all studied estimators (H_E_, H_O_, F_ST_, D), measured as correlation, average relative difference and slope of the regression line when comparing the biased estimators to the according gold standard. The effect was already present when using a very small reference set of only one sequenced individual per population. Imputation also performed better than simple filtering strategies based on the array data alone. However, when using imputation for ascertainment bias reduction care has to be taken in designing an evenly spaced reference panel to not introduce a new bias towards variation present in the reference panel while missing variants of other populations. We also suggest using a larger reference panel than the one which was available for this study to achieve better results. Additionally, we observed an effect of array design on imputation accuracy as discovery populations showed a higher imputation accuracy than non-discovery populations. This should be taken into account when designing studies based on imputed SNPs by choosing an appropriate genotyping array for the intended study populations.

## Supplementary Information

**Additional file 1: Supplementary File 1**. Accession Information of raw data per sample.

**Additional file 2: Supplementary File 2**. Supplementary Methods.

**Additional file 3: Figure S1**. Recall rates for samples which were genotyped as well as sequenced per SNP (A; B) and per animal (C; D); before (A; C; red) and after (B; D; blue) correction of potential reference allele switches in the genotype data.

**Additional file 4: Figure S2**. Development of the per-animal imputation accuracy with an increasing number of reference animals per population. A – scenario randSamp_5_50; B – scenario randPop_5_50; C – scenario minPop_5_50; D – scenario maxPop_5_50. Individuals are grouped on whether they belong to the population which contains reference individuals, was used as for SNP discovery or none of them (application). the lines show the trend of the median.

**Additional file 5: Figure S3**. Development of correlations within population group (r), slope and mean overestimation of the regression lines for H_E_ and H_O_ estimates and different reference panel strategies. The intended value for unbiasedness and minimum variance is marked as dense black horizontal line. Note that the case without imputation is consistent with zero reference samples.

**Additional file 6: Figure S4**. Development of correlation within population group (A), slope (B) and intercept (C) of the regression lines for D and F_ST_ when distributing the reference samples equally over all populations (allPop_74_740). The intended value for unbiasedness and minimum variance is marked as dense black horizontal line. Note that the case without imputation is consistent with zero reference samples.

**Additional file 7: Figure S5**. Development of correlation within population group (r), slope and mean overestimation of the regression lines for Nei’s Distance (D) and F_ST_ estimates and different reference panel strategies. The intended value for unbiasedness and minimum variance is marked as dense black horizontal line. Note that the case without imputation is consistent with zero reference samples.

**Additional file 8: Figure S6**. Distribution of DR2 values by chromosome and reference set. Note that outliers are not shown due to a large number of underlying values.

**Additional file 9: Figure S7**. Two-dimensional distributions of DR2 values vs. MAF by chromosome when imputed with the reference set 74_1perLine. The red line represents the median within 0.05 MAF bins.

**Additional file 10: Figure S8**. Effect of pooled sequencing and the correction factor of Futschik and Schlötterer [50] on expected heterozygosity (HE) and ascertainment bias. A – HE estimated from array positions of the sequencing data vs. HE directly estimated from array data. The color indicates the state before and after correcting the pooled sequence estimates and the accordingly colored solid lines the group specific regression lines while the black solid line indicates the line of identity in all three plots. The plot therefore shows the magnitude of the bias introduced by pooled sequencing and the according effect of the correction factor. B – HE estimated from the array data vs. HE estimated from the complete sequence data. The color again shows the values before and after implementing the correction of the pooled sequence estimates. While the solid regression line and dense circles indicate the individually sequenced samples, the dashed regression lines and triangles indicate pooled sequenced samples. The plot therefore shows the combined effect of ascertainment bias and pooled sequencing bias. C – HE estimated from array positions of the sequencing data vs. HE estimated from all positions of the sequencing data. The plot therefore shows the pure ascertainment bias.

**Additional file 11: Figure S9**. Effect of pooled sequencing on the expression of the ascertainment bias in Nei’s standard genetic distance (D). The biased D was either estimated directly from the array genotypes (D.arr, pooled bias + ascertainment bias) or from the array positions of the sequencing data (D.arr.seq, pure ascertainment bias), while the estimates from the complete sequence were assumed to be the true estimates. The black solid line represents the line of identity, solid colored regression lines and dense points represent estimates between individually sequenced populations and dashed lines and triangles represent estimates between two populations of which at least one was pooled sequenced.

**Additional file 12: Figure S10**. Effect of pooled sequencing on the expression of the ascertainment bias in Wright’s fixation index (F_ST_). The biased F_ST_ was either estimated directly from the array genotypes (FST.arr, pooled bias + ascertainment bias) or from the array positions of the sequencing data (FST.arr.seq, pure ascertainment bias), while the estimates from the complete sequence were assumed to be the true estimates. The black solid line represents the line of identity, solid colored regression lines and dense points represent estimates between individually sequenced populations and dashed lines and triangles represent estimates between two populations of which at least one was pooled sequenced.

**Additional file 13: Figure S11**. Per animal imputation accuracies (r) for the array to sequence imputation from leave-one-out validation. Results are shown for four reference sets and three chromosomes. Results are further separated on whether the accuracy for the animal was derived by being the test sample in the leave-one-out validation run or the animal was not part of the reference set at all (only possible for commercial samples with multiple individual sequences per population and not for the scenario 158_all). Colour further indicates whether the test sample of the according validation run was a commercial or a non-commercial chicken. Detailed information about the implications can be found in Supplementary File 2.

**Additional file 14: Table S1**. Quantiles of theoretical imputation accuracies (DR2) by reference set.

## Data Availability

Raw sequencing and genotyping data were previously published by different studies. The repository information for each sample can be found in Supplementary File 1. All datasets generated by analyses during this study from the raw data are additionally available from the corresponding author on reasonable request.

## Abbreviations

D: Nei’s standard genetic distance
F_ST_: Wright’s fixation index
H_E_: Expected heterozygosity
H_O_: Observed heterozygosity
MAF: Minor allele frequency
r: Pearson correlation
SNP: Single nucleotide polymorphism
WGS: Whole genome sequencing
