# Supplementary material for: How imputation can mitigate SNP ascertainment Bias
Source: BMC Genomics. 2021 May 12;22:340. doi: 10.1186/s12864-021-07663-6 (PMC8114708; doi:10.1186/s12864-021-07663-6)
Supplement: Supplementary file 2 — Additional file 2: Supplementary File 2. Supplementary Methods. [file 12864_2021_7663_MOESM2_ESM.pdf]

How Imputation Can Mitigate SNP Ascertainment Bias

Supplementary File 2: Supplementary Methods and Results

Johannes Geibel[12*], Christian Reimer[12], Torsten Pook[12], Steffen Weigend[23], Annett Weigend[3] & Henner Simianer[12]

[1]University of Goettingen, Department of Animal Sciences, Animal Breeding and Genetics Group, Albrecht-Thaer-Weg 3, 37075 Göttingen, Germany; johannes.geibel@uni-goettingen.de; creimer@gwdg.de; torsten.pook@uni-goettingen.de; hsimian@gwdg.de

[2]University of Goettingen, Center for Integrated Breeding Research, Albrecht-Thaer-Weg 3, 37075 Göttingen, Germany

[3]Friedrich-Loeffler-Institut, Institute of Farm Animal Genetics, Höltystrasse 10, 31535 Neustadt-Mariensee, Germany; steffen.weigend@fli.de; annett.weigend@fli.de

[*] johannes.geibel@uni-goettingen.de (Corresponding Author)

Calling of WGS SNPs

Alignment of the raw sequencing reads and SNP calling was conducted for individual and pooled sequenced data following GATK best practices [1, 2]. In detail, raw WGS reads were aligned against the latest chicken reference genome GRCg6a [3] using bwa-0.7.17 mem [4], duplicates were marked using gatk-4.1.2 MarkDuplicates [5], base qualities were recalibrated using gatk-4.1.2 BaseRecalibrator [5] and SNPs were called via assigning per sample reference confidence scores with gatk-4.1.2 HaplotypeCaller [5] in GVCF mode and then genotyped across samples via gatk-4.1.2 GenotypeGVCFs [5]. Raw variants were finally filtered using gatk-4.1.2 VariantRecalibrator [5], which filtered the called SNPs by a machine learning approach, which uses both, a set of previously known (low confidence needed) and a set of highly reliable (assumed to be true) variants as training resources [6]. The source for known SNPs (prior 2) provided to VariantRecalibrator was ENSEMBL (release 96; [7]) and the SNPs of the Axiom™ Genome-Wide Chicken Array were defined as the true training set (prior 15). The algorithm was trained on the quality parameters DP, QD, FS, SOR, ReadPosRankSum, MQ and MQRank-Sum and the number of Gaussians was limited to 6. Filters were set to recover 98 % of the training SNPs in the filtered set, which resulted in a Transition/Transversion ratio of 2.55 for known SNPs, and a Transition/Transversion ratio of 2.30 for novel SNPs. This resulted in 20,829,081 biallelic SNPs on chromosomes 1 - 28 which were used in further analyses.

Additionally, all individual sequences were genotyped for the positions of the Affymetrix Axiom™ 580k Genome-Wide Chicken Array [8] by running gatk-4.1.2 HaplotypeCaller [5] in GENOTYPE_GIVEN_ALLELES mode.

Generation of genotype set

To ensure compatibility between Array- and WGS data, the latest Affymetrix annotation file [9], which is based on galGal5, was lifted over to galGal6 using gatk-4.1.2 LiftoverVcf [5] under usage of the according UCSC chain file [10, 11]. Switches of reference and alternative allele were present in the SCDP data set due to the lift over or the introduction of major allele coding by previous use of plink 1.9 [12] to manage the SCDP data set. To correct for this problem, reference and alternative allele code were switched for SNPs with homozygous recall rates < 0.5 across individuals who were genotyped and sequenced. This massively increased recall rates for the SNPs (Figure S 1).

Only SNPs with known autosomal position, call rates > 0.95 and genotype recall rates > 0.95 were further considered. MAF filters were later used when subsampling the different sets and thus not considered in this step. Further, missing genotypes were imputed using Beagle 5.0 [13] with ne=1000 [14] and the genetic map taken from Groenen *et al.* [15]. Note that Beagle 5.0 was used even though Beagle 5.1 already existed, as Pook *et al.* [14] found Beagle 5.0 to result in slightly fewer phasing errors for plant and livestock data sets. This resulted in a final set of 1,566 animals from 74 populations (18 - 37 animals per population) and 462,549 autosomal SNPs, further referred to as the **genotype set**.

As Malomane *et al.* [16] described LD-based pruning as an effective filtering strategy to minimize the impact of ascertainment bias in SNP array data, the genotype set was additionally LD pruned using plink 1.9 [12] with --indep 50 5 2 flag. This reduced the genotype set to 136,755 SNPs (30 %) and will be referred to as **pruned genotype set**.

Effect of pooling bias

Pooled sequencing is assumed to be a cost-effective approach for the estimation of population allele frequencies. However, it comes along with a series of error sources as varying sequencing error rates, reference allele preferential bias, within-sample amplification bias, and cross-sample amplification bias [17]. The thereby introduced bias should be minimal if pool size and sequencing depth are large [18]. However, as the pools used in our study are relatively small (9 – 11 individuals) and some are additionally weakly covered (15 – 70 X per population; ~1.5 – 7X per individual), we identified a slight but unneglectable underestimation of H_E (Figure S 8 A), D (Figure S 9) and F_{ST} (Figure S 10) compared to individually sequenced data. This therefore would have affected implications on ascertainment bias, as the bias introduced by pooling could not have been separated from the ascertainment bias (Figure S 8 B, Figure S 8 C, Figure S 9, Figure S 10). The problem is additionally severe as the commercial populations which are closely related to the discovery populations of the array [8] are individually sequenced and the bias due to pooling is therefore strongly confounded with one major source of ascertainment bias, the use of arrays in non-target populations [19, 20]. An expected clearly stronger ascertainment bias for populations closely related to the populations used for SNP discovery (Figure S 8 C) was therefore masked by the pooling bias (Figure S 8 B). To account for the pooling bias, H_E for pooled sequences was multiplied with the correction factor $\frac{n}{n-1}$, introduced by Futschik and Schlötterer [21], where n is the number of haplotypes in the pool. This slightly, but not completely, corrected the bias (Figure S 8 A). Due to the still remaining bias and as it was not possible to include this correction factor also into the estimates of D and F_{ST}, we separately evaluate the effects of imputation on WGS scale for the individually sequenced individuals and all individuals. In the case of F_{ST}, in contrast to the pooled bias (Figure S 10)

and in accordance with Albrechtsen *et al.* [22], ascertainment bias showed close to no effect. We, therefore, do not include F_{ST} in the discussion on sequence level at all.

Imputation accuracy

In silico array to genotype set

Per-animal imputation accuracies (r) were already high (median = 0.94) when using one reference individual per population (allPop_74). Increasing the number of reference individuals subsequently increased the accuracy up to 0.99 for 10 reference individuals per population (allPop_740). The accuracy was consistently higher for individuals which were part of the discovery population (Figure 5).

Accuracies were lower for the other strategies, mainly due to fewer reference individuals (maximal 50). However, Figure S 2 indicates that the best imputation accuracies were achieved for populations which contained at least one reference individual. The discovery populations showed slightly to strongly increased accuracies compared to the application populations for all scenarios besides the scenario where the reference populations were chosen to be maximum distant to the discovery population (scenario maxPop_5_50; Figure S 2). Randomly choosing reference individuals across the complete population set (scenario randSamp_5_50; Figure S 2) resulted in the overall best imputation accuracies. When assigning the reference samples to randomly chosen populations in blocks of five samples per population (scenario randPop_5_50, Figure S 2), the accuracies for the reference populations were only slightly increased, while accuracies for discovery- and application populations dropped massively. Interestingly, the accuracies for application populations were higher in scenario minPop_5_50

(Figure S 2), in which the reference populations were chosen according to their minimum distance to the discovery population, than with the random reference population selection from scenario randPop_5_50. A selection of reference populations to be maximum distant to the discovery population (scenario maxPop_5_50, Figure S 2) performed overall worst.

Genotype set to WGS

Imputation accuracy for the genotype set to WGS imputation was assessed by performing leave-one-out validation for three reference set strategies (74_1perLine, 98_5perLine, 158_all) and three chromosomes (chr1, chr10, chr25). For this, each reference sample was deleted from the reference set for one single run of imputation (test sample). The correlation between imputed and true genotypes for the test sample was then recorded. Additionally, track was kept of the correlation between the imputed and true genotypes for the commercial samples which were sequenced, but not used in the according reference set. As for 68 non-commercial samples, there was only one sequenced individual per population, the removal of this single individual from the reference set led to a reference set without any individual from the according population. We therefore expected a strong downward bias for imputation accuracy in those lines. For the commercial samples, there were either two lines with one sample each in the reference set from each population (74_1perLine) or multiple individuals per line (98_5perLine, 158_all) in the reference set, meaning that at least one relatively closely related individual remained in the reference set. The bias should therefore be less strong than for the non-commercial samples. To acccount for this, we additionally evaluated a fourth reference set (71_1commLine) where we kept only one white layer, brown layer and broiler line each in the reference set. Finally, we used the internal Beagle quality measure, the dosage r-squared (DR2) [57] to evaluate theoretical per-SNP imputation accuracy.

Figure S 11 summarises the results of the leave-one-out validation. The expected bias is present but numerically small for the commercial test samples with mean Δr of 0.011 (chr1) 0.009 (chr10) and 0.025 (chr25) between the reference sets 71_1commLine and 74_1perLine. Note, that a quantification for the non-commercial lines is not possible from our data. Adding commercial samples to the reference set (74_1perLine to 158_all) only increased accuracies of the commercial samples. When evaluating DR2 values, the smallest reference panel (74_1perLine) showed a median DR2 of 0.93 with 5 % and 25 % quantiles being 0.73 and 0.88. Increasing the number of reference samples from the commercial populations to 158_all increased the 5% quantile of DR2 by 0.05 while increasing all higher quantiles only by 0.01 (Table S 1). Note that chromosomes 16, 22 and 25 clearly showed a higher proportion of badly imputed SNPs than the other chromosomes (Figure S 6, Figure S 7) which goes along with clearly lower per-SNP imputation accuracies for chr25 compared to chr1 and chr10 in the leave-one-out validation (Figure S 11). This lower accuracy for the three chromosomes was expected, as they are known for complex structural rearrangements (e.g. the MHC complex on chr16).

References

1. DePristo MA, Banks E, Poplin R, Garimella KV, Maguire JR, Hartl C, et al. A framework for variation discovery and genotyping using next-generation DNA sequencing data. Nat. Genet. 2011;43:491. doi:10.1038/ng.806.
2. van der Auwera GA, Carneiro MO, Hartl C, Poplin R, Del Angel G, Levy-Moonshine A, et al. From FastQ data to high confidence variant calls: The Genome Analysis Toolkit best practices pipeline. Curr Protoc Bioinformatics. 2013;43:11.10.1-11.10.33. doi:10.1002/0471250953.bi1110s43.

3. Genome Reference Consortium GRCg6a. GRCg6a chicken reference genome. 2018. http://hgdownload.soe.ucsc.edu/goldenPath/galGal6/bigZips/galGal6.fa.gz. Accessed 9 Apr 2019.
4. Li H. BWA: Burrow Wheelers Aligner; 2014.
5. McKenna A, Hanna M, Banks E, Sivachenko A, Cibulskis K, Kernytsky A, et al. The Genome Analysis Toolkit: A MapReduce framework for analyzing next-generation DNA sequencing data. Genome Res. 2010;20:1297–303. doi:10.1101/gr.107524.110.
6. Broad Institute. GATK User Guide. 2018. https://software.broadinstitute.org/gatk/documentation/. Accessed 20 Mar 2018.
7. Cunningham F, Achuthan P, Akanni W, Allen J, Amode MR, Armean IM, et al. Ensembl 2019. Nucleic Acids Res. 2018;47:D745-D751. doi:10.1093/nar/gky1113.
8. Kranis A, Gheyas AA, Boschiero C, Turner F, Le Yu, Smith S, et al. Development of a high density 600K SNP genotyping array for chicken. BMC Genomics. 2013;14:59. doi:10.1186/1471-2164-14-59.
9. Affymetrix Inc. Axiom_GW_GTChicken Annotations. 22.06.2017. https://sec-assets.thermofisher.com/TFS-Assets/LSG/Support-Files/Axiom_GW_GT_Chicken.na35.annot.csv.zip. Accessed 14 Apr 2019.
10. Hinrichs AS, Karolchik D, Baertsch R, Barber GP, Bejerano G, Clawson H, et al. The UCSC Genome Browser Database: Update 2006. Nucleic Acids Res. 2006;34:D590-8. doi:10.1093/nar/gkj144.
11. UCSC. galGal5ToGalGal6 chain file. 2018. http://hgdownload.soe.ucsc.edu/goldenPath/galGal5/liftOver/galGal5ToGalGal6.over.chain.gz. Accessed 15 Apr 2019.

12. Chang CC, Chow CC, Tellier LC, Vattikuti S, Purcell SM, Lee JJ. Second-generation PLINK: Rising to the challenge of larger and richer datasets. Gigascience. 2015;4:7. doi:10.1186/s13742-015-0047-8.

13. Browning BL, Zhou Y, Browning SR. A One-Penny Imputed Genome from Next-Generation Reference Panels. Am. J. Hum. Genet. 2018;103:338–48. doi:10.1016/j.ajhg.2018.07.015.

14. Pook T, Mayer M, Geibel J, Weigend S, Cavero D, Schoen CC, Simianer H. Improving Imputation Quality in BEAGLE for Crop and Livestock Data. G3. 2019:g3.400798.2019. doi:10.1534/g3.119.400798.

15. Groenen MAM, Wahlberg P, Foglio M, Cheng HH, Megens H-J, Crooijmans RPMA, et al. A high-density SNP-based linkage map of the chicken genome reveals sequence features correlated with recombination rate. Genome Res. 2009;19:510–9.

16. Malomane DK, Reimer C, Weigend S, Weigend A, Sharifi AR, Simianer H. Efficiency of different strategies to mitigate ascertainment bias when using SNP panels in diversity studies. BMC Genomics. 2018;19:22. doi:10.1186/s12864-017-4416-9.

17. Chen X, Listman JB, Slack FJ, Gelernter J, Zhao H. Biases and Errors on Allele Frequency Estimation and Disease Association Tests of Next-Generation Sequencing of Pooled Samples. Genet. Epidemiol. 2012;36:549–60. doi:10.1002/gepi.21648.

18. Schlötterer C, Tobler R, Kofler R, Nolte V. Sequencing pools of individuals [mdash] mining genome-wide polymorphism data without big funding. Nat. Rev. Genet. 2014;15:749–63.

19. Nielsen R. Population genetic analysis of ascertained SNP data. Hum. Genomics. 2004;1:1.

20. Geibel J, Reimer C, Weigend S, Weigend A, Pook T, Simianer H. How Array Design Affects SNP Ascertainment Bias. bioRxiv. 2019:833541. doi:10.1101/833541.

21. Futschik A, Schlötterer C. The Next Generation of Molecular Markers From Massively Parallel Sequencing of Pooled DNA Samples. Genetics. 2010;186:207–18. doi:10.1534/genetics.110.114397.

22. Albrechtsen A, Nielsen FC, Nielsen R. Ascertainment biases in SNP chips affect measures of population divergence. Mol. Biol. Evol. 2010;27:2534–2547.
