# Supplementary figures and images for: How imputation can mitigate SNP ascertainment Bias
Source: BMC Genomics. 2021 May 12;22:340. doi: 10.1186/s12864-021-07663-6 (PMC8114708; doi:10.1186/s12864-021-07663-6)

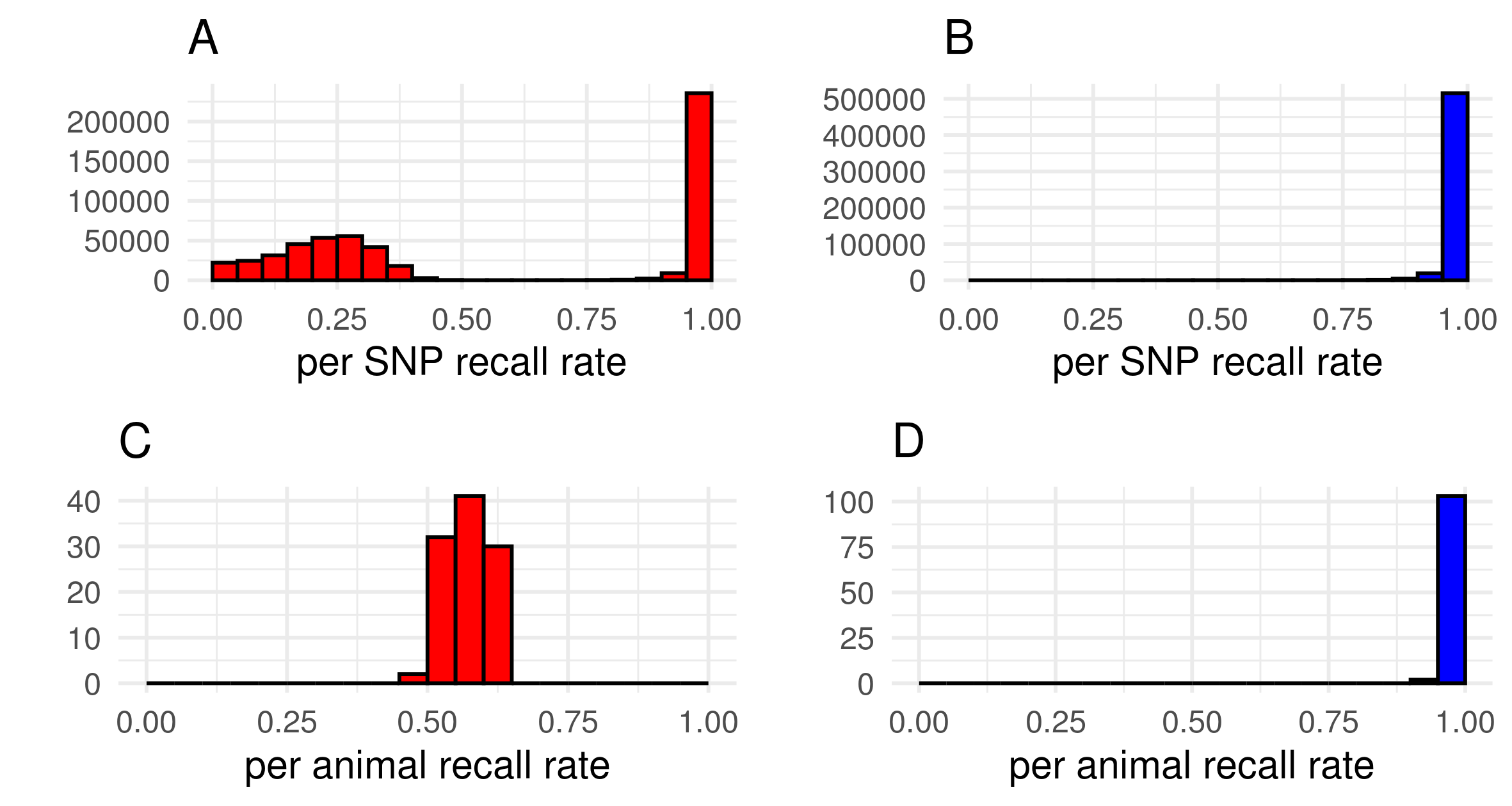

Supplement: Supplementary file 3 — Additional file 3: Figure S1. Recall rates for samples which were genotyped as well as sequenced per SNP (A; B) and per animal (C; D); before (A; C; red) and after (B; D; blue) correction of potential reference allele switches in the genotype data. [file 12864_2021_7663_MOESM3_ESM.tiff]

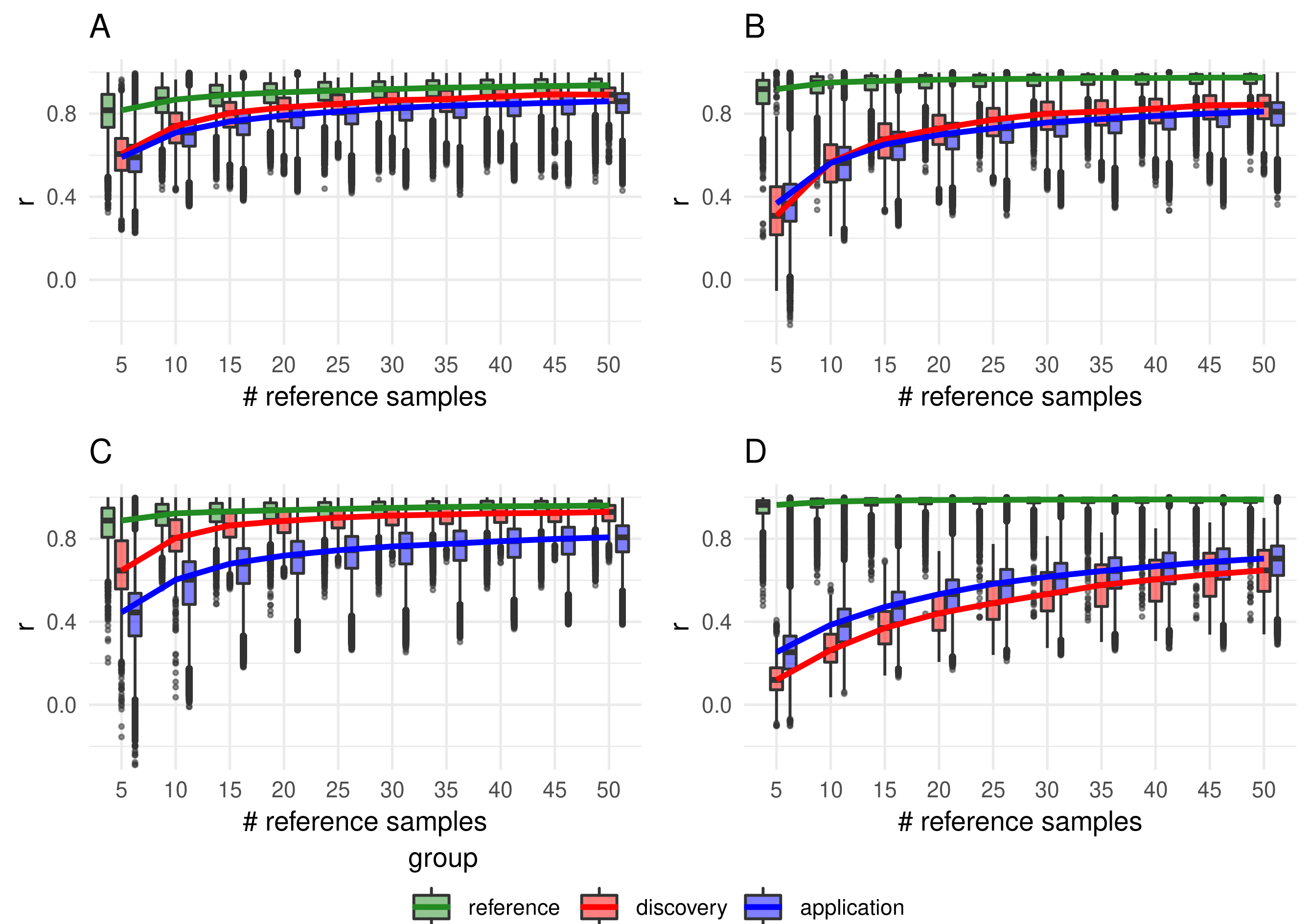

Supplement: Supplementary file 4 — Additional file 4: Figure S2. Development of the per-animal imputation accuracy with an increasing number of reference animals per population. A – scenario randSamp_5_50; B – scenario randPop_5_50; C – scenario minPop_5_50; D – scenario maxPop_5_50. Individuals are grouped on whether they belong to the population which contains reference individuals, was used as for SNP discovery or none of them (application). the lines show the trend of the median. [file 12864_2021_7663_MOESM4_ESM.tiff]

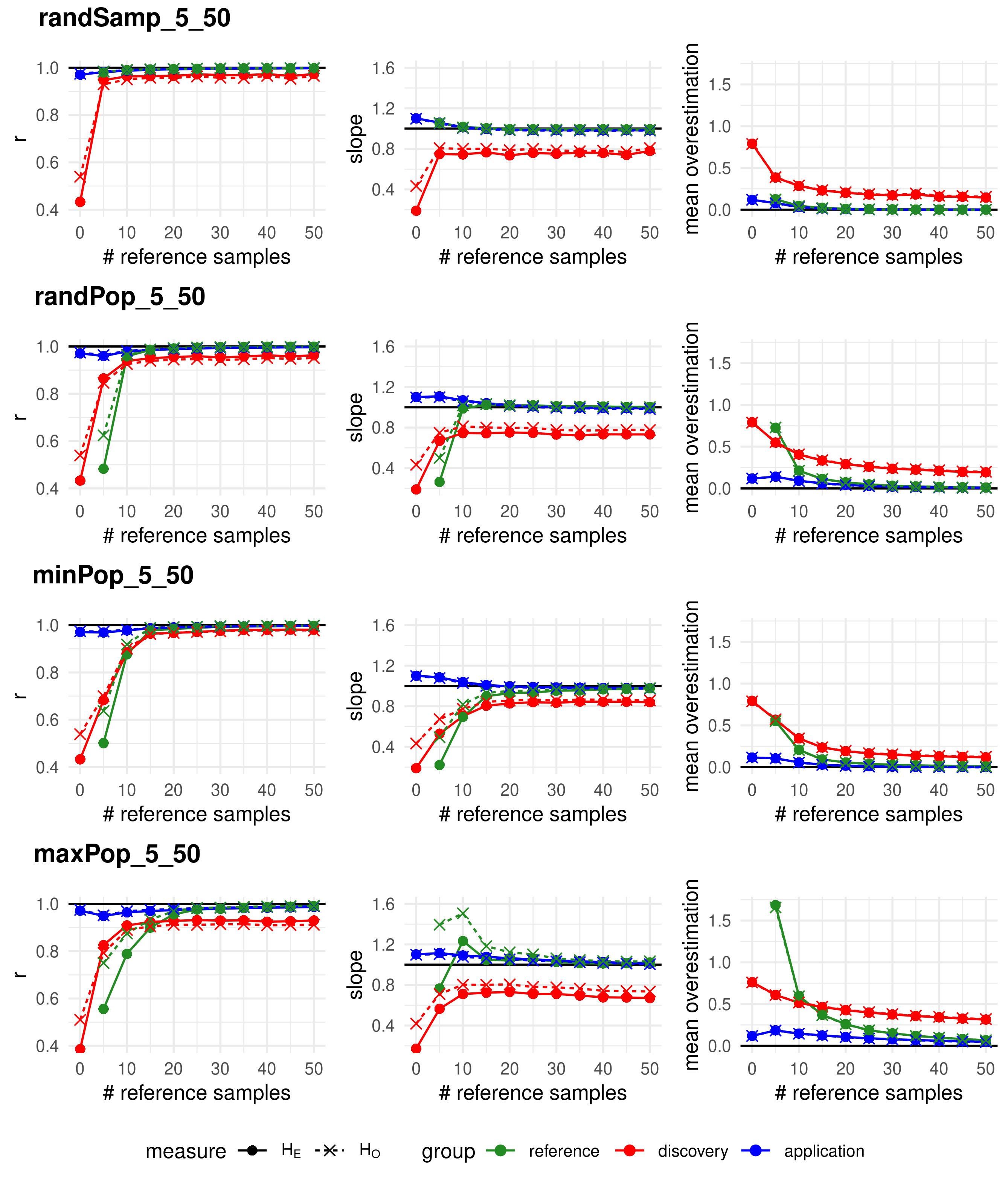

Supplement: Supplementary file 5 — Additional file 5: Figure S3. Development of correlations within population group (r), slope and mean overestimation of the regression lines for HE and HO estimates and different reference panel strategies. The intended value for unbiasedness and minimum variance is marked as dense black horizontal line. Note that the case without imputation is consistent with zero reference samples. [file 12864_2021_7663_MOESM5_ESM.tiff]

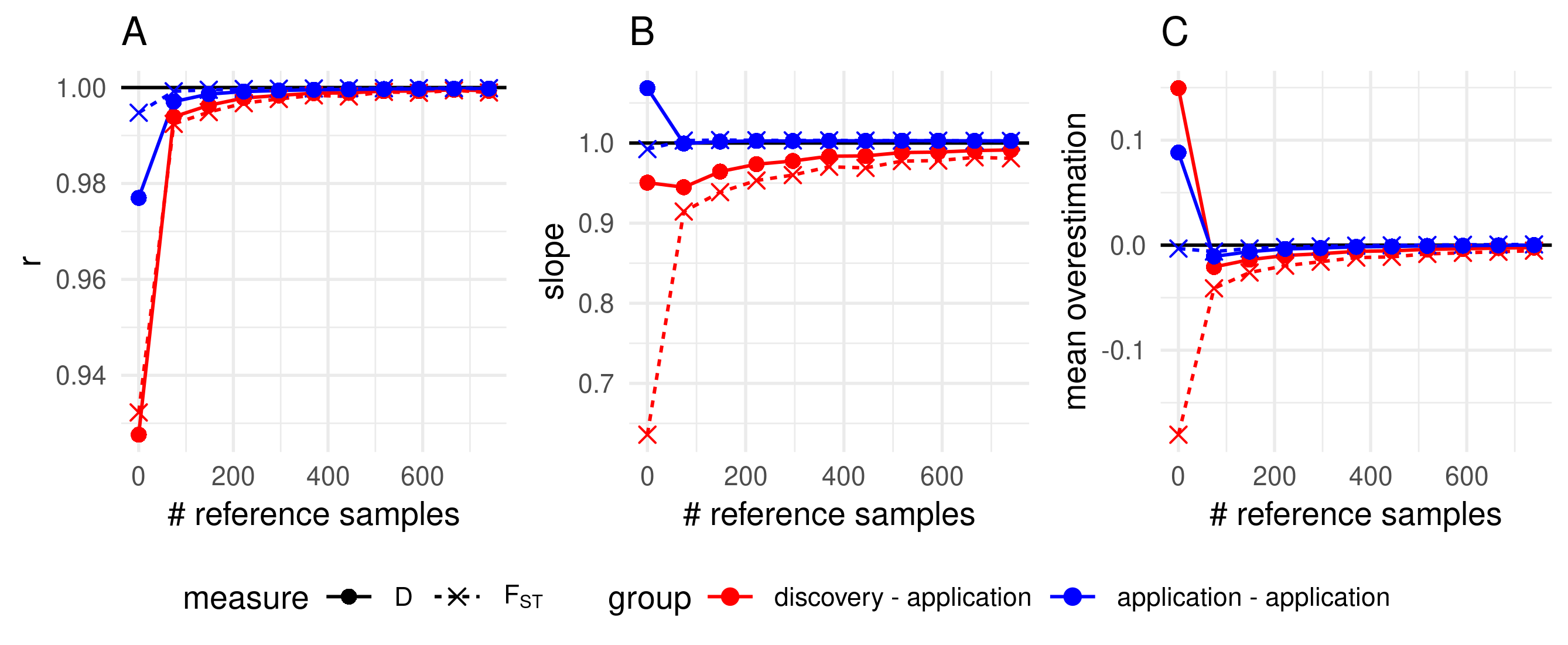

Supplement: Supplementary file 6 — Additional file 6: Figure S4. Development of correlation within population group (A), slope (B) and intercept (C) of the regression lines for D and FST when distributing the reference samples equally over all populations (allPop_74_740). The intended value for unbiasedness and minimum variance is marked as dense black horizontal line. Note that the case without imputation is consistent with zero reference samples. [file 12864_2021_7663_MOESM6_ESM.tiff]

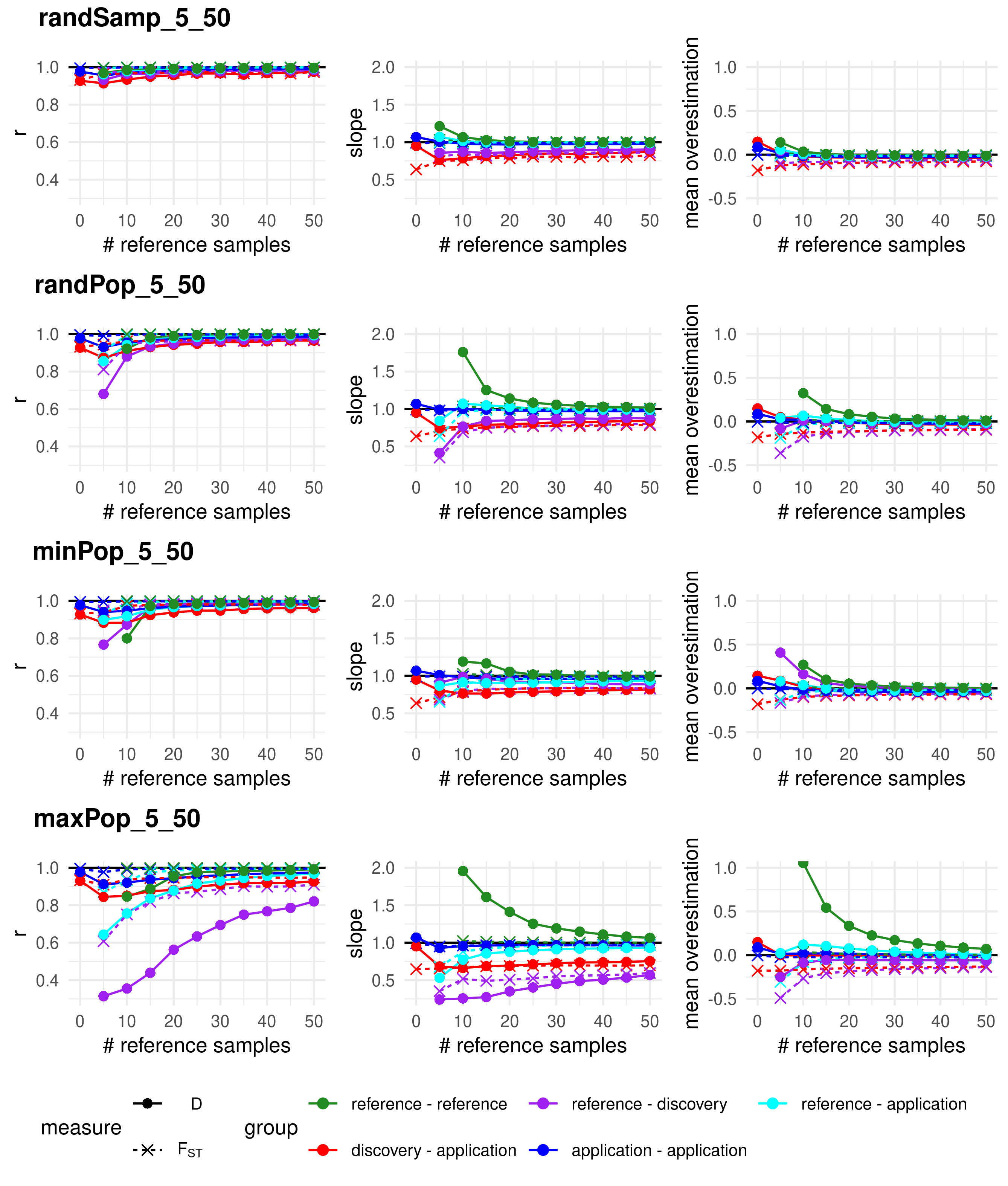

Supplement: Supplementary file 7 — Additional file 7: Figure S5. Development of correlation within population group (r), slope and mean overestimation of the regression lines for Nei’s Distance (D) and FST estimates and different reference panel strategies. The intended value for unbiasedness and minimum variance is marked as dense black horizontal line. Note that the case without imputation is consistent with zero reference samples. [file 12864_2021_7663_MOESM7_ESM.tiff]

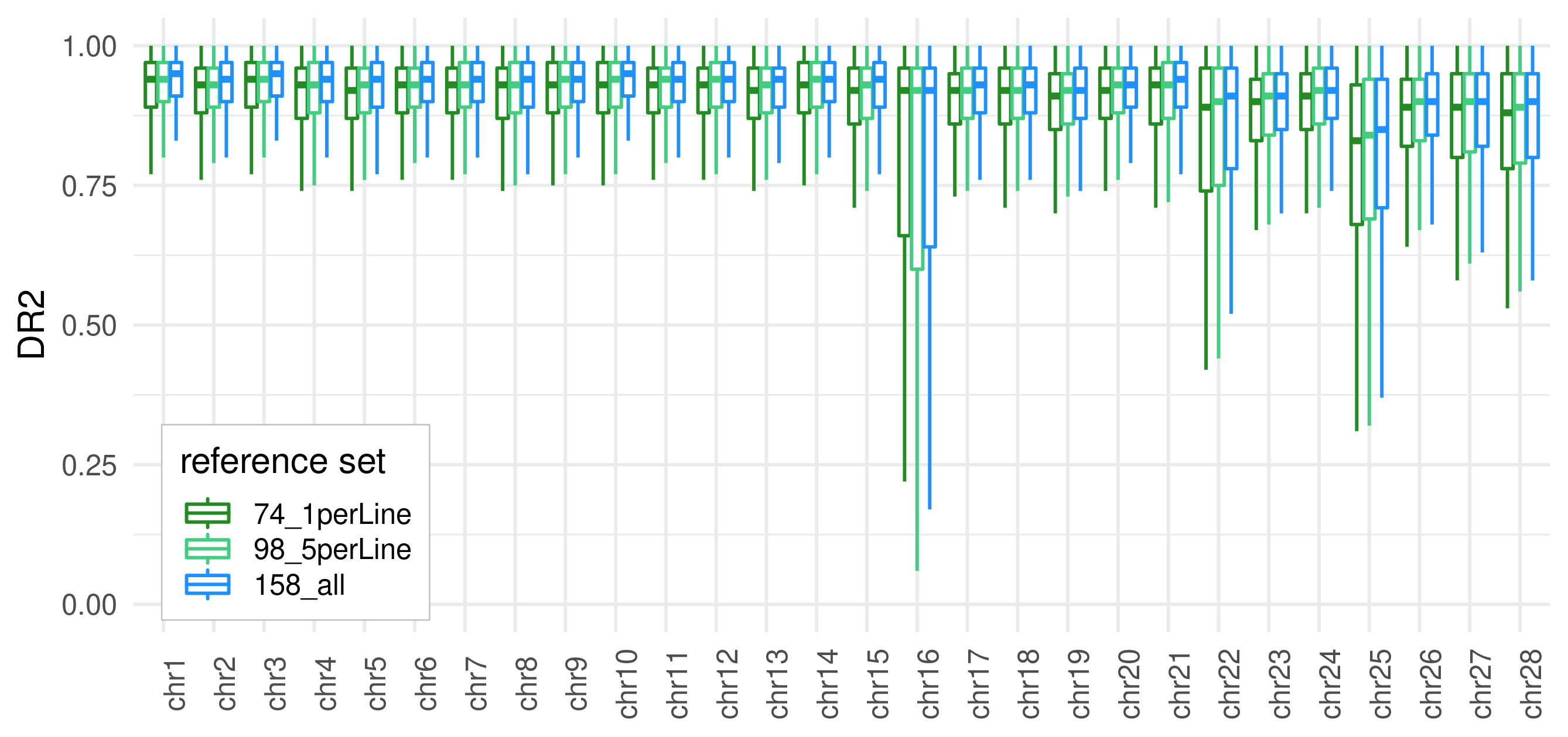

Supplement: Supplementary file 8 — Additional file 8: Figure S6. Distribution of DR2 values by chromosome and reference set. Note that outliers are not shown due to a large number of underlying values. [file 12864_2021_7663_MOESM8_ESM.tiff]

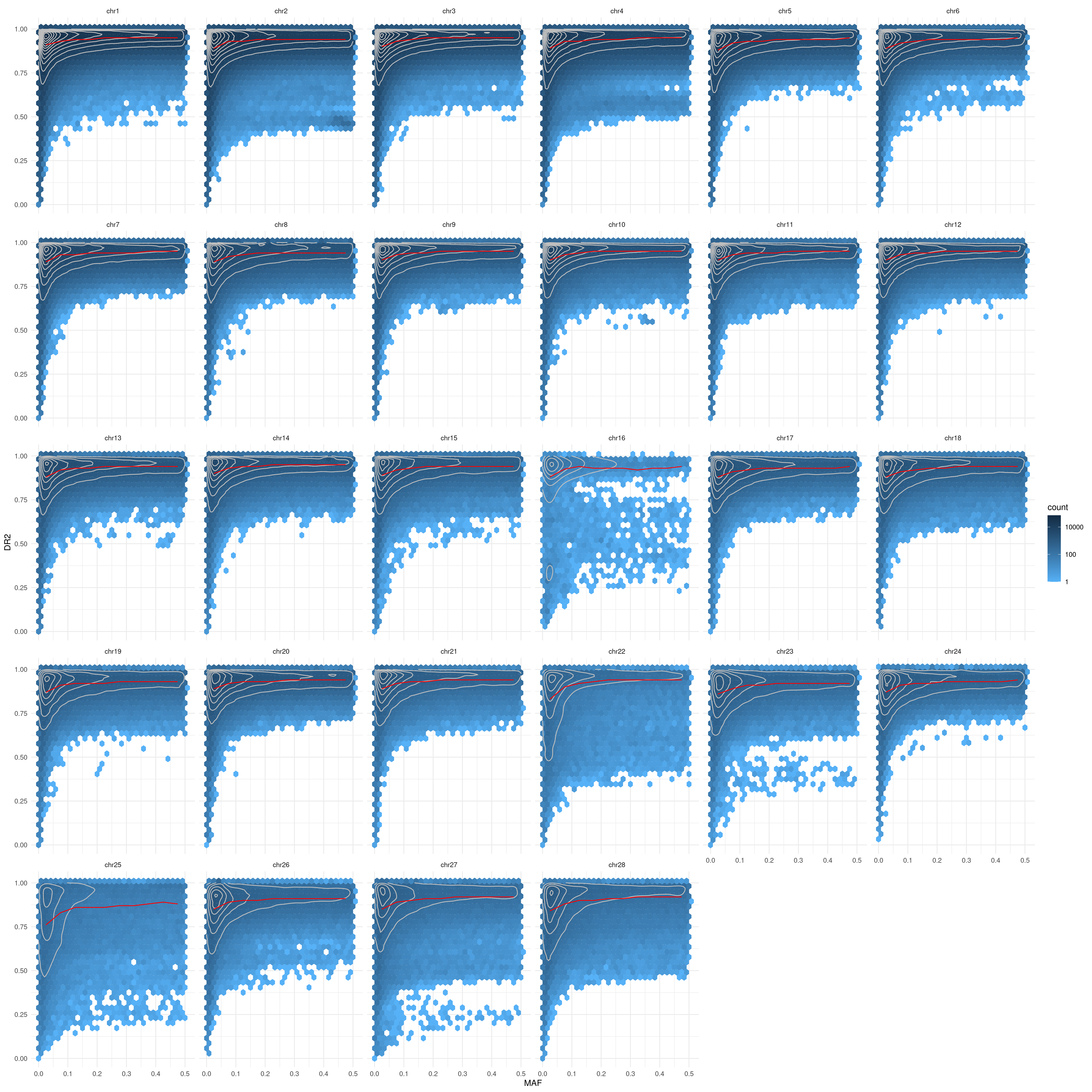

Supplement: Supplementary file 9 — Additional file 9: Figure S7. Two-dimensional distributions of DR2 values vs. MAF by chromosome when imputed with the reference set 74_1perLine. The red line represents the median within 0.05 MAF bins. [file 12864_2021_7663_MOESM9_ESM.tiff]

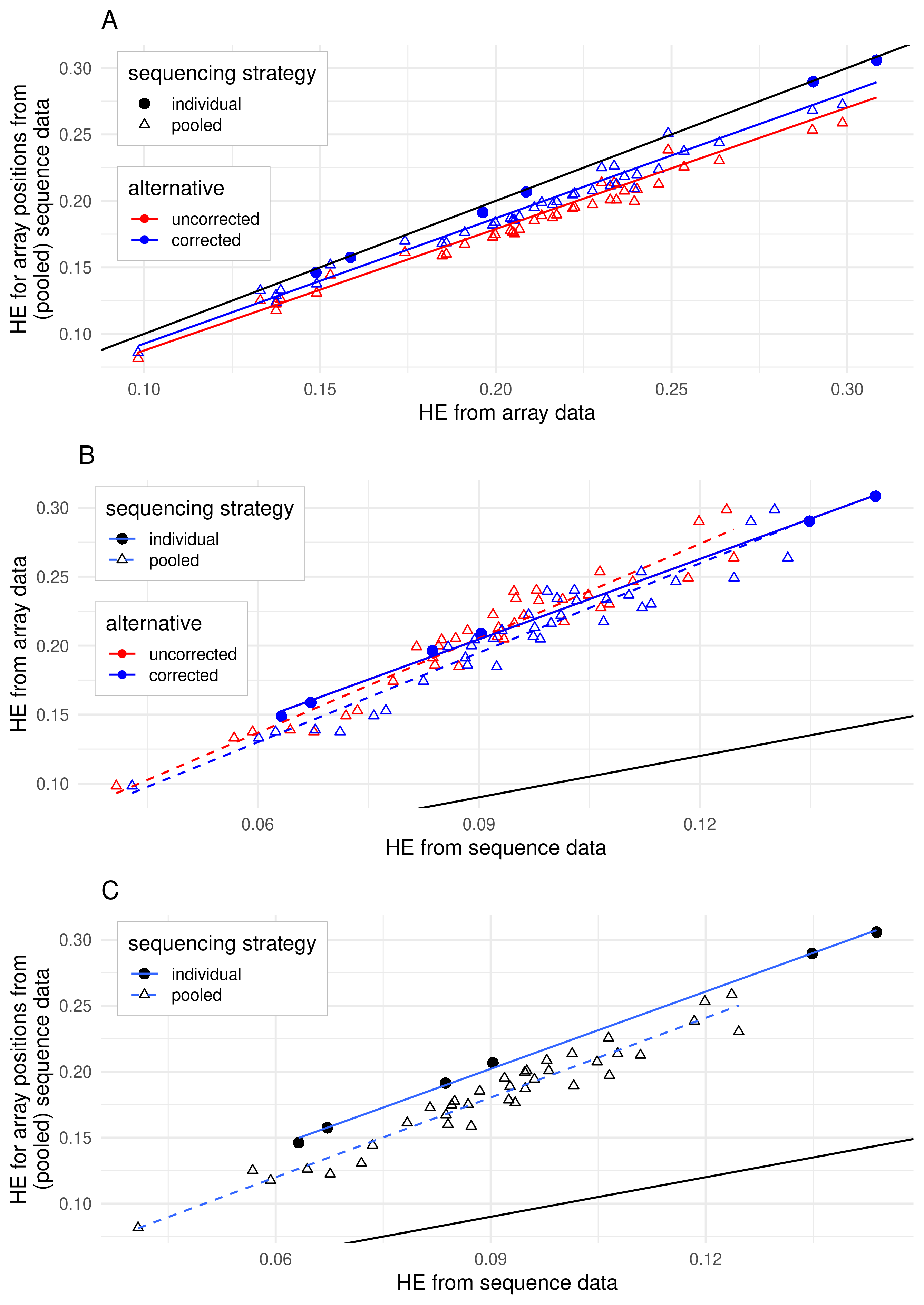

Supplement: Supplementary file 10 — Additional file 10: Figure S8. Effect of pooled sequencing and the correction factor of Futschik and Schlötterer [50] on expected heterozygosity (HE) and ascertainment bias. A – HE estimated from array positions of the sequencing data vs. HE directly estimated from array data. The color indicates the state before and after correcting the pooled sequence estimates and the accordingly colored solid lines the group specific regression lines while the black solid line indicates the line of identity in all three plots. The plot therefore shows the magnitude of the bias introduced by pooled sequencing and the according effect of the correction factor. B – HE estimated from the array data vs. HE estimated from the complete sequence data. The color again shows the values before and after implementing the correction of the pooled sequence estimates. While the solid regression line and dense circles indicate the individually sequenced samples, the dashed regression lines and triangles indicate pooled sequenced samples. The plot therefore shows the combined effect of ascertainment bias and pooled sequencing bias. C – HE estimated from array positions of the sequencing data vs. HE estimated from all positions of the sequencing data. The plot therefore shows the pure ascertainment bias. [file 12864_2021_7663_MOESM10_ESM.tiff]

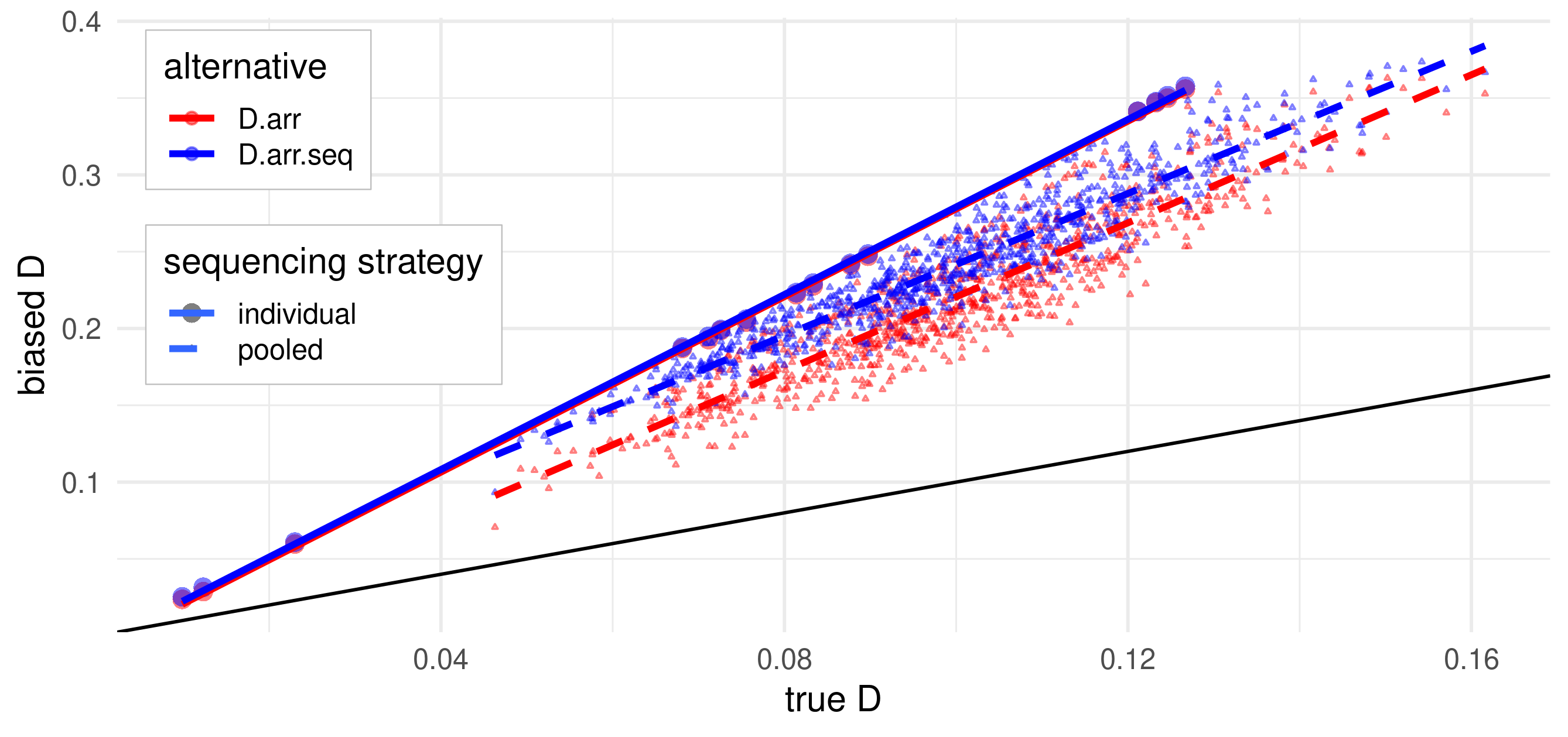

Supplement: Supplementary file 11 — Additional file 11: Figure S9. Effect of pooled sequencing on the expression of the ascertainment bias in Nei’s standard genetic distance (D). The biased D was either estimated directly from the array genotypes (D.arr, pooled bias + ascertainment bias) or from the array positions of the sequencing data (D.arr.seq, pure ascertainment bias), while the estimates from the complete sequence were assumed to be the true estimates. The black solid line represents the line of identity, solid colored regression lines and dense points represent estimates between individually sequenced populations and dashed lines and triangles represent estimates between two populations of which at least one was pooled sequenced. [file 12864_2021_7663_MOESM11_ESM.tiff]

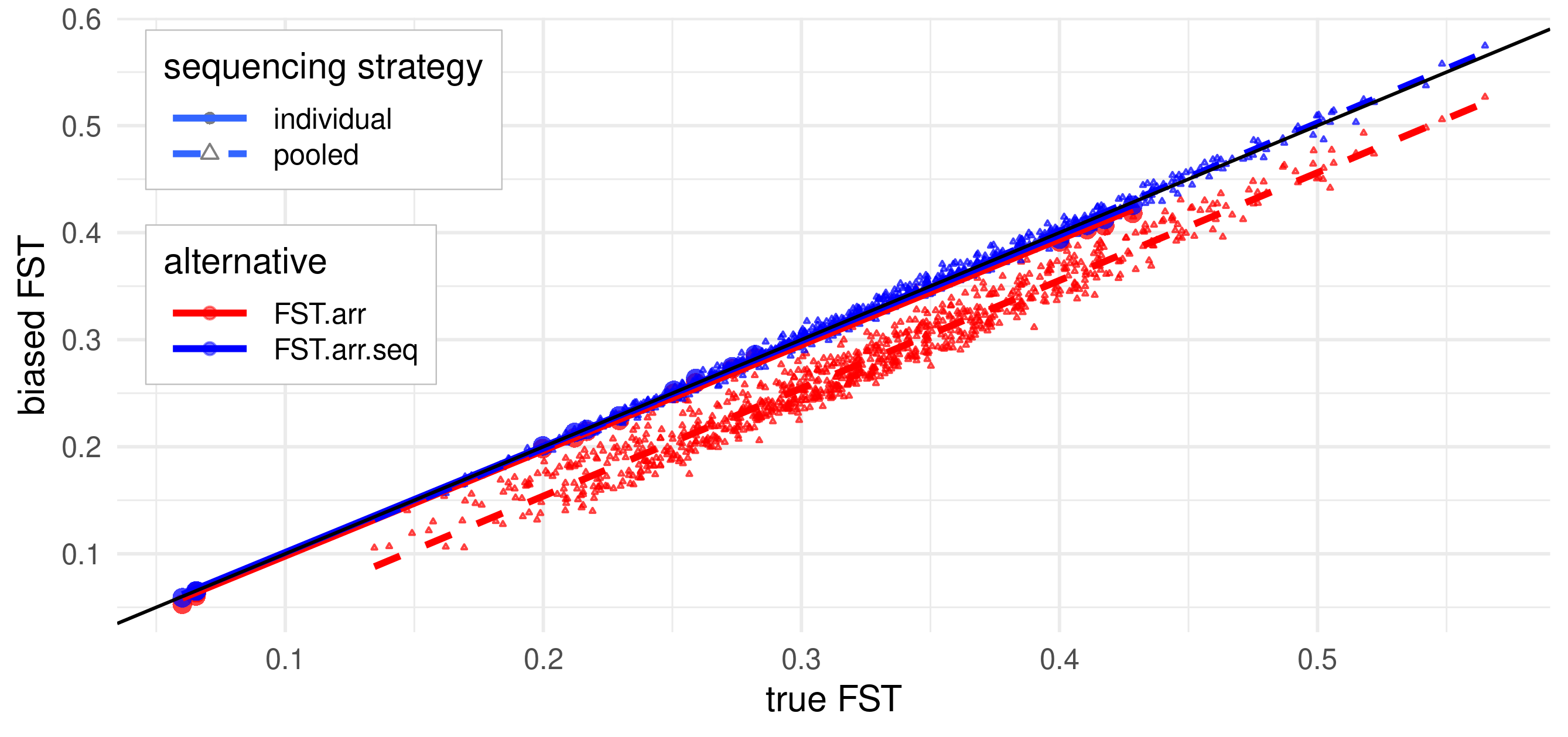

Supplement: Supplementary file 12 — Additional file 12: Figure S10. Effect of pooled sequencing on the expression of the ascertainment bias in Wright’s fixation index (FST). The biased FST was either estimated directly from the array genotypes (FST.arr, pooled bias + ascertainment bias) or from the array positions of the sequencing data (FST.arr.seq, pure ascertainment bias), while the estimates from the complete sequence were assumed to be the true estimates. The black solid line represents the line of identity, solid colored regression lines and dense points represent estimates between individually sequenced populations and dashed lines and triangles represent estimates between two populations of which at least one was pooled sequenced. [file 12864_2021_7663_MOESM12_ESM.tiff]

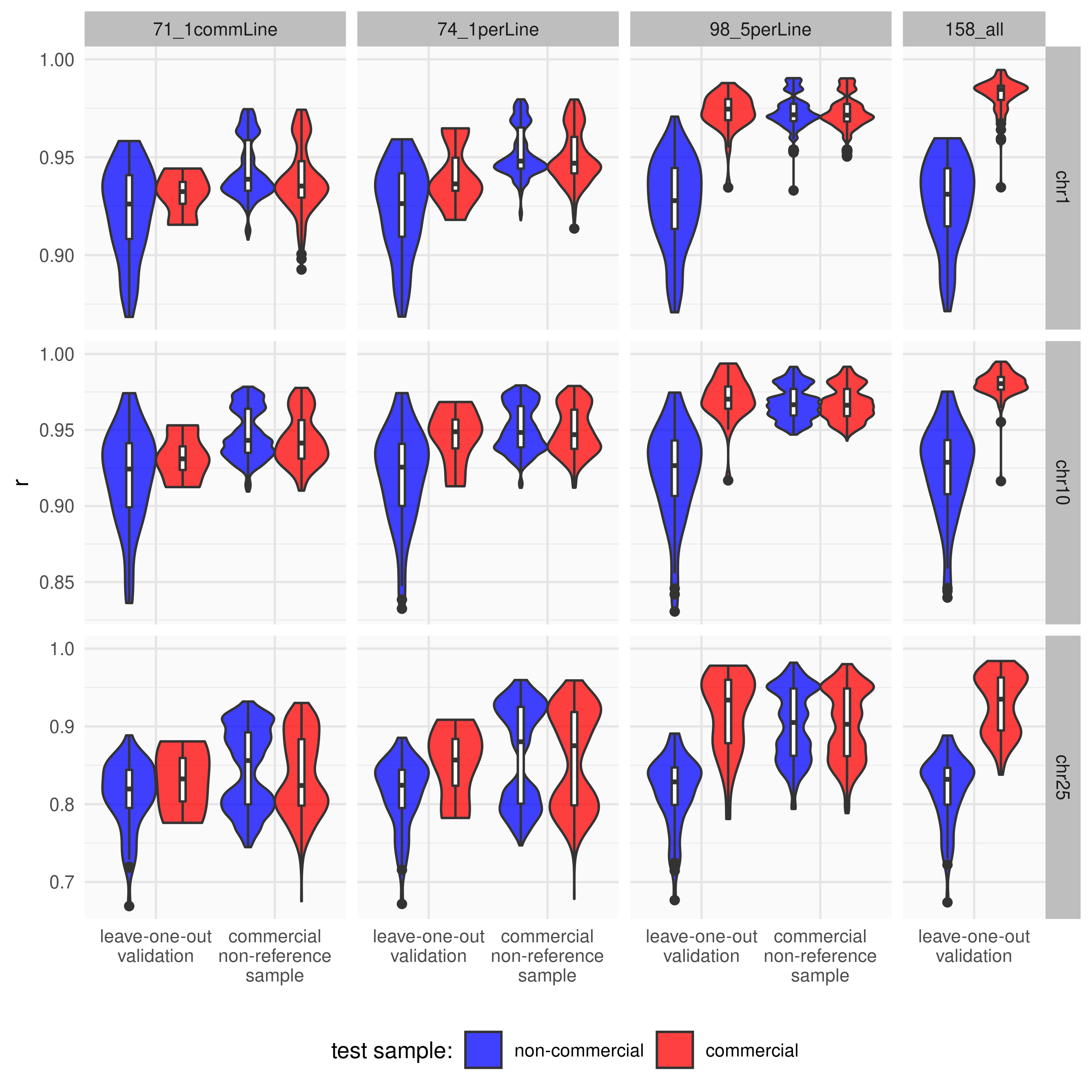

Supplement: Supplementary file 13 — Additional file 13: Figure S11. Per animal imputation accuracies (r) for the array to sequence imputation from leave-one-out validation. Results are shown for four reference sets and three chromosomes. Results are further separated on whether the accuracy for the animal was derived by being the test sample in the leave-one-out validation run or the animal was not part of the reference set at all (only possible for commercial samples with multiple individual sequences per population and not for the scenario 158_all). Colour further indicates whether the test sample of the according validation run was a commercial or a non-commercial chicken. Detailed information about the implications can be found in Supplementary File 2. [file 12864_2021_7663_MOESM13_ESM.tiff]
