# Supplementary material for: How imputation can mitigate SNP ascertainment Bias
Source: BMC Genomics. 2021 May 12;22:340. doi: 10.1186/s12864-021-07663-6 (PMC8114708; doi:10.1186/s12864-021-07663-6)
Supplement: Supplementary file 14 — Additional file 14: Table S1. Quantiles of theoretical imputation accuracies (DR2) by reference set. [file 12864_2021_7663_MOESM14_ESM.docx]

Table S 1: Quantiles of theoretical imputation accuracies (DR2) by reference set

| Reference set | DR2_Q05 | DR2_Q25 | DR2_median | DR2_mean | DR2_Q75 |
| --- | --- | --- | --- | --- | --- |
| 74_1perLine | 0.73 | 0.88 | 0.93 | 0.90 | 0.96 |
| 80_2perLine | 0.74 | 0.88 | 0.93 | 0.91 | 0.96 |
| 86_3perLine | 0.74 | 0.88 | 0.93 | 0.91 | 0.96 |
| 92_4perLine | 0.74 | 0.88 | 0.93 | 0.91 | 0.96 |
| 98_5perLine | 0.74 | 0.89 | 0.93 | 0.91 | 0.97 |
| 158_all | 0.79 | 0.90 | 0.94 | 0.92 | 0.97 |
